# Mucosal-associated invariant T cells recognize a tumor-derived metabolite in the DNA synthesis pathway

**DOI:** 10.3389/fimmu.2026.1797918

**Published:** 2026-06-24

**Authors:** Yanqi Xue, Yuka Yamada, Rikako Suzuki, Brenda Luong, Shotaro Fujii, Keisuke Nakata, Masatomo Takahashi, Yoshihiro Izumi, Chihiro Fukui, Ryosuke Takasaki, Francois Legoux, Shinsuke Inuki, Daisuke Motooka, Emi Ito, Bridget L. Stocker, Mattie S. M. Timmer, Takashi Shimizu, Yasumasa Matsuoka, Jun’ichi Mano, Koji Tamada, Makoto Furutani-Seiki, Kei Sakamoto, Koh-Hei Sonoda, Olivier Lantz, Sho Yamasaki, Kensuke Shibata

**Affiliations:** 1 Department of Ophthalmology, Graduate School of Medical Sciences, Kyushu University, Fukuoka, Japan; 2 Department of Microbiology and Immunology, Graduate School of Medicine, Yamaguchi University, Ube, Japan; 3 School of Chemical and Physical Sciences, Victoria University of Wellington, Wellington, New Zealand; 4 Division of Metabolomics, Medical Research Center for High Depth Omics, Medical Institute of Bioregulation, Kyushu University, Fukuoka, Japan; 5 Graduate School of Pharmaceutical Sciences, Kyoto University, Kyoto, Japan; 6 INSERM ERL1305, CNRS UMR6290, Institut de Génétique et Développement de Rennes, Université de Rennes, Rennes, France; 7 Graduate School of Biomedical Sciences, Tokushima University, Tokushima, Japan; 8 NGS core facility, Bioinformatics Center, Research Institute for Microbial Diseases, Osaka University, Suita, Japan; 9 Department of Molecular Immunology, Research Institute for Microbial Diseases, Osaka University, Suita, Japan; 10 Joint Faculty of Veterinary Medicine, Laboratory of Veterinary Public Health, Yamaguchi University, Yamaguchi, Japan; 11 Advanced Technology Institute, Yamaguchi University, Yamaguchi, Japan; 12 Science Research Center, Organization for Research Initiatives, Yamaguchi University, Yamaguchi, Japan; 13 Department of Immunology, Graduate School of Medicine, Yamaguchi University, Ube, Japan; 14 The Research Institute for Cell Design Medical Science, Yamaguchi University, Ube, Japan; 15 Systems Biochemistry in Pathology and Regeneration, Graduate School of Medicine, Yamaguchi University, Ube, Japan; 16 Institut Curie, Paris Sciences et Lettres Université, Inserm U932, Immunity and Cancer, Paris, France; 17 Laboratory of Molecular Immunology, Immunology Frontier Research Center, Osaka University, Suita, Japan; 18 Division of Molecular Immunology, Medical Mycology Research Center, Chiba University, Chiba, Japan; 19 Center for Advanced Modalities and DDS, Osaka University, Suita, Japan; 20 Center for Infectious Disease Education and Research, Osaka University, Suita, Japan; 21 Department of Visual Regeneration, Graduate School of Medical Sciences, Kyushu University, Fukuoka, Japan; 22 Department of Molecular and Cellular Physiology, Graduate School of Medicine, Yamaguchi University, Ube, Japan

**Keywords:** cancer, DNA synthesis, ligand, metabolite, T cells

## Abstract

Mucosal-associated invariant T (MAIT) cells recognize metabolite-derived ligands, but the identity of such ligands in the context of cancer remains poorly defined. Herein, we demonstrate that the tumor-derived metabolite 5-formyl tetrahydrofolate (5-formyl THF), which is an intermediate of the folate metabolism pathway, induces T cell receptor (TCR)-dependent activation of mouse mucosal-associated invariant T (MAIT) cells. The activity was weaker than that of a potent MAIT cell agonist 5-(2-oxopropylideneamino)-6-d-ribitylaminouracil (5-OP-RU). *Amino methyltransferase* (*Amt*), an enzyme that is necessary for the generation of 5-formyl THF from tumor cells, was essential for the activation of mouse MAIT cells. Genetic deletion of *Amt* in the tumor resulted in impaired activation of mouse MAIT cells. In contrast, *Amt* overexpression in the tumor led to an enhancement in the agonistic activity of the MAIT cells. The introduction of mouse MAIT TCRs to primary T cells from MAIT cell-deficient mice also conferred reactivity to 5-formyl THF. Using single-cell TCR sequencing analysis, a 5-formyl THF-reactive MAIT cell clonotype was identified in humans. These results demonstrate that 5-formyl THF is the first tumor-derived ligand recognized by MAIT cells in both humans and mice. However, the functional relevance of this pathway *in vivo* remains to be determined.

## 1 Introduction

Cancer is a leading cause of death worldwide (1). Infiltrated cytotoxic T cell frequencies positively correlate with prolonged survival rates for various cancer patients (2). These cytotoxic T cells can be categorized into two subsets: major histocompatibility complex (MHC) class I-restricted, and MHC class I-non-restricted T cells. MHC class I-restricted T cells, which recognize tumor-associated peptides, have been extensively studied for applications in cancer immunotherapy (3). Notwithstanding, the meta-analysis of gene expression in tumor-associated cells revealed that better survival rates were seen in patients with high expression levels of *KLRB1* encoding CD161 (4), a marker for MHC class I-non-restricted T cell subsets that include MHC-class-I-related protein 1 (MR1)-restricted αβ T cells (5) and those that express a semi-invariant T cell receptor (TCR), termed mucosal-associated invariant T (MAIT) cells (6). Non-MAIT MR1-restricted αβ T cells and MAIT cells have been shown to infiltrate tumors and exhibit tumor-killing activity (5, 7–9), yet the pro-tumor activity of MAIT cells has been reported in tumor mouse models (10, 11). Given that MAIT cell functions can be modulated in a T cell receptor (TCR)-dependent manner, identification of MR1-restricted αβ T cell ligands represents an essential first step toward understanding MAIT cell biology in the tumor microenvironment.

MAIT cells are the most dominant MR1-restricted αβ T cell subset in both humans and mice (12). The majority of MAIT cells express a semi-invariant TCRα chain which is encoded by rearranged genes: *TRAV1-2/TRAJ33/TRAC* for humans; *Trav1/Traj33/Trac* for mice (12, 13). The MAIT TCRα chain is preferentially heterodimerized with specific TCRβ chains harboring TRBV6 and TRBV20–1 in humans and Trbv13 and Trbv19 in mice (14–17). Through their TCRs, MAIT cells recognize microbial riboflavin precursor derivatives, 5-OP-RU and 5-(2-oxoethylideneamino)-6-D-ribitylaminouracil (5-OE-RU) (18). Indeed, tumor-associated bacteria from colorectal cancer (CRC) patients activated MAIT cells in a TCR-dependent mechanism (19). The presence of putative tumor-derived ligands captured by MR1 has also been reported (10, 20–22). Recently, adenine (8-(9H-purin-6-yl)-2-oxa-8-azabicyclo [3.3.1] nona-3,6-diene-4,6-dicarbaldehyde [M_3_Ade]) was identified as the tumor-derived ligand for a subset of MR1-restricted αβ T cells, although M3Ade did not activate the major MR1-restricted αβ T cell subset, MAIT cells (5). Thus, to date, the structures of tumor-derived metabolites that are recognized by MAIT cells have not been determined.

In the present study, we identified 5-formyl tetrahydrofolate (5-formyl THF), a tumor-derived metabolite generated during DNA synthesis, as an agonistic MAIT cell ligand. The introduction of mouse MAIT TCRs to primary T cells from MAIT cell-deficient *Traj33*^–/–^ mice conferred reactivity to 5-formyl THF. Using single-cell RNA sequencing coupled with TCR analysis then allowed for the identification of a human MAIT cell clonotype that recognizes 5-formyl THF. 5-Formyl THF is the first tumor-derived MAIT cell ligand to date.

## 2 Results

### 2.1 Identification of a mouse MAIT cell clone that recognizes non-microbial ligands

**Figure 1:**
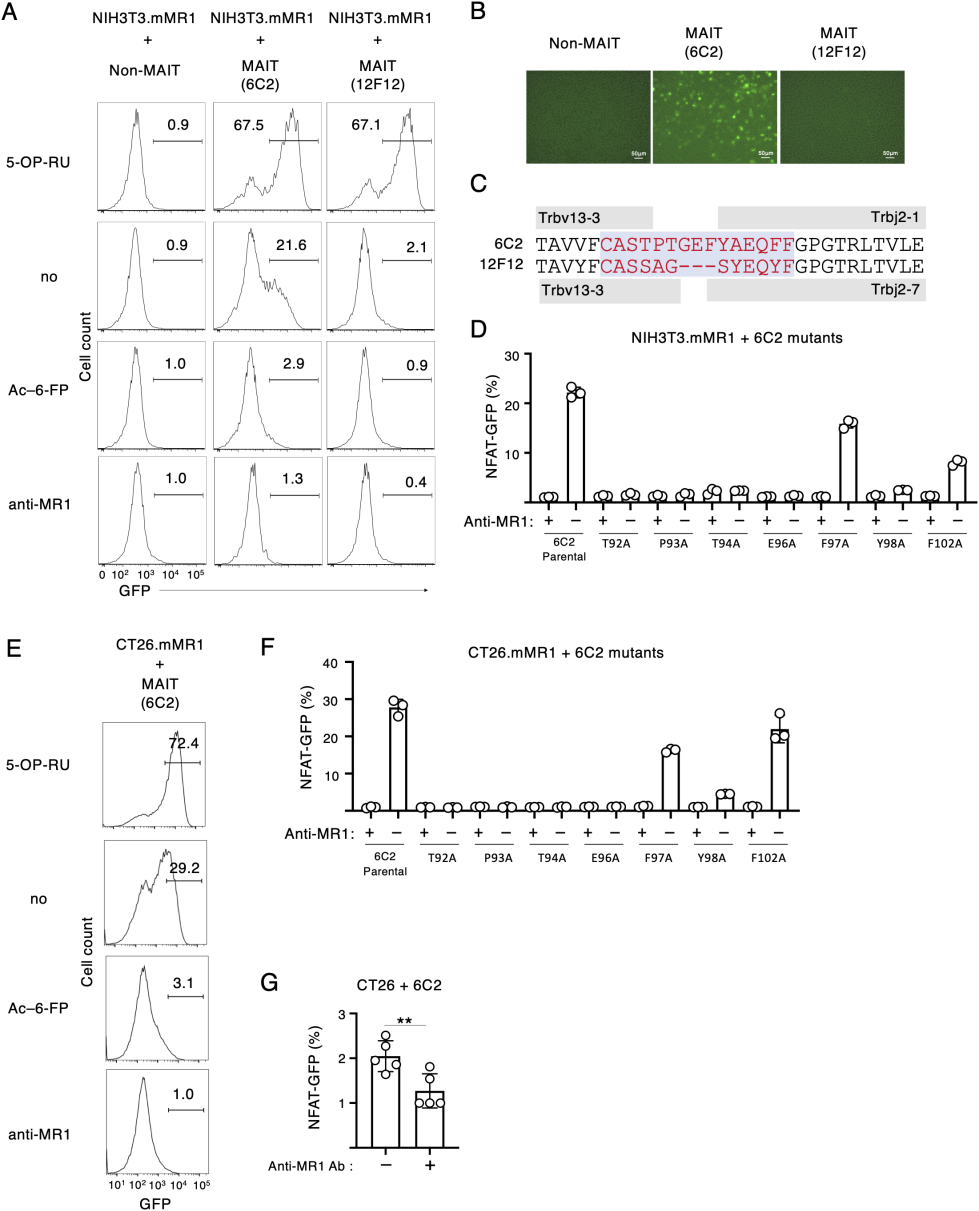
Mouse MAIT cell line 6C2 recognizes non-microbial ligands. **(A, B, D)** NFAT-GFP expressions of MAIT reporter cell lines (6C2, 12F12) and a non-MAIT cell reporter cell line were analyzed using a flow cytometer **(A, D)** and a BZ-X700 confocal microscope **(B)** after co-culture with NIH3T3.mMR1 cells in the presence of Ac-6-FP (100 μM), 5-OP-RU (10 nM) and an anti-MR1 blocking antibody. **(B)** Scale bars show 50 μm length. **(E–G)** NFAT-GFP expressions of 6C2 cells and its mutants were analyzed using a flow cytometer after co-culture with CT26.mMR1 cells **(E, F)** or CT26 cells **(G)** in the presence of Ac-6-FP (100 μM) or 5-OP-RU (10 nM) or an anti-MR1 blocking antibody. **(A, D, E–G)** Percentages of NFAT-GFP^+^ cells were calculated after gating on 7AAD-negative TCRβ^+^ cells. **(A, E)** Numbers in the histogram show the percentage of GFP^+^ cells. **(D, F)** Bar graphs show percentages of NFAT-GFP^+^ cells in 6C2 cells (parental) and their mutants (T92A, P93A, T94A, E96A, F97A, Y98A, F102A). Data are means ± SEM from three biological replicates per group. **(G)** Bar graph shows percentages of NFAT-GFP^+^ cells in 6C2 cells. Data are means ± SEM from five biological replicates per group. Statistical significance was determined by unpaired two-tailed Student’s *t*-test (** *p* < 0.01). **(C)** Illustration shows comparison of junctional amino acid sequences including CDR3β regions (red) between 6C2 and 12F12 TCRs by ClustalW program (https://www.genome.jp/tools-bin/clustalw). Data are representative of two independent experiments.

To search for MAIT cell ligands, we generated two mouse MAIT TCR-expressing GFP reporter cell lines (6C2 TCR: Trav1/Traj33/Trac and Trbv13-3/Trbd2/Trbj2-1/Trbc1, 12F12 TCR: Trav1/Traj33/Trac and Trbv13-3/Trbd2/Trbj2-7/Trbc1) (15). After co-culture with mouse MR1-overexpressing fibroblastic cells (referred to as NIH3T3.mMR1 cells hereafter) (23), the mouse MAIT cell lines 6C2 and 12F12, but not the non-MR1-restricted αβ T cell line (24), expressed GFP upon stimulation with the most potent agonist 5-OP-RU (**Figure 1A**). This MAIT cell ligand screening system allowed us to determine the presence of cognate MAIT cell ligands by analyzing GFP reporter fluorescence. Notably, after co-culture, a substantial increase of GFP expression in 6C2 cells without the addition of exogenous agonists was observed (**Figures 1A, B**). GFP expression in 6C2 cells was abolished in the presence of either an anti-MR1 blocking antibody or the known antagonist, acetyl-6-formylpterin (Ac-6-FP) (**Figure 1A**; **Supplementary Figure S1A**), suggesting TCR-dependent activation through putative ligands. This finding was consistent with previous studies that demonstrated that the 6C2 clone was able to recognize non-microbial ligands (13, 25, 26). To determine which amino acids of the 6C2 TCR were responsible for the observed activity, the TCRβ sequences of 6C2 and 12F12 were compared (their TCRα chains were identical). Comparative analysis of the TCRβ sequences revealed differences in the complementary determining region 3β (CDR3β) but no other regions (**Figure 1C**). To test the importance of the CDR3β for agonistic activity, 6C2-derived mutants harboring single amino acid substitutions in the CDR3β were generated. All mutants expressed comparable levels of TCR on the cell surface (**Supplementary Figure S1B**). Using these cell lines, reporter activity was analyzed after co-culture with NIH3T3.mMR1 cells. For this analysis, in each cell line, the gating strategy that showed 1% in groups treated with an anti-MR1 blocking antibody (**Supplementary Figure S1A**) was applied to non-treated (**Figure 1D**) and 5-OP-RU-treated groups (**Supplementary Figure S1C**). The substitution of amino acids T^92^, P^93^, T^94^, E^96^, and Y^98^, but not F^97^ or F^102^, led to the complete abrogation of GFP expression (**Figure 1D**). In response to 5-OP-RU, complete loss of GFP expression in the E^96^ mutant was observed (**Supplementary Figure S1C**). These results suggest that 6C2 cells recognize non-microbial ligands derived from NIH3T3.mMR1 cells in a TCR-dependent mechanism and that the TCRβ chain is critical for agonistic activity of MAIT cells, as exemplified previously for microbial ligands (27, 28).

### 2.2 The mouse MAIT cell clone 6C2 recognizes tumor-derived ligands

Recent studies have shown that tumor-infiltrating MAIT cells in CRCs have an anti-tumor activity in both humans and mice (7, 8, 29, 30). Accordingly, we sought to determine whether CRCs had the potential to activate mouse MAIT cells. To address this, we generated mouse MR1-overexpressing colorectal tumor CT26 cells (referred to as CT26.mMR1 cells hereafter). The CT26.mMR1 cells also induced the TCR-dependent activation of 6C2 cells (**Figure 1E**). As was observed for the NIH3T3.mMR1 cells (**Figure 1D**), particular CDR3β amino acids, such as T92, T94, E96, and Y98, were responsible for the activation of 6C2 cells (**Figure 1F**). The agonistic activity of 6C2 cells was also observed using CT26 cells without MR1 overexpression, albeit with lower potency (**Figure 1G**). These results suggest that CT26 cells activate 6C2 cells, and that MR1 overexpression on CT26 cells increases the agonistic activity.

### 2.3 5-Formyl THF is a tumor-derived ligand for mouse MAIT cells

**Figure 2:**
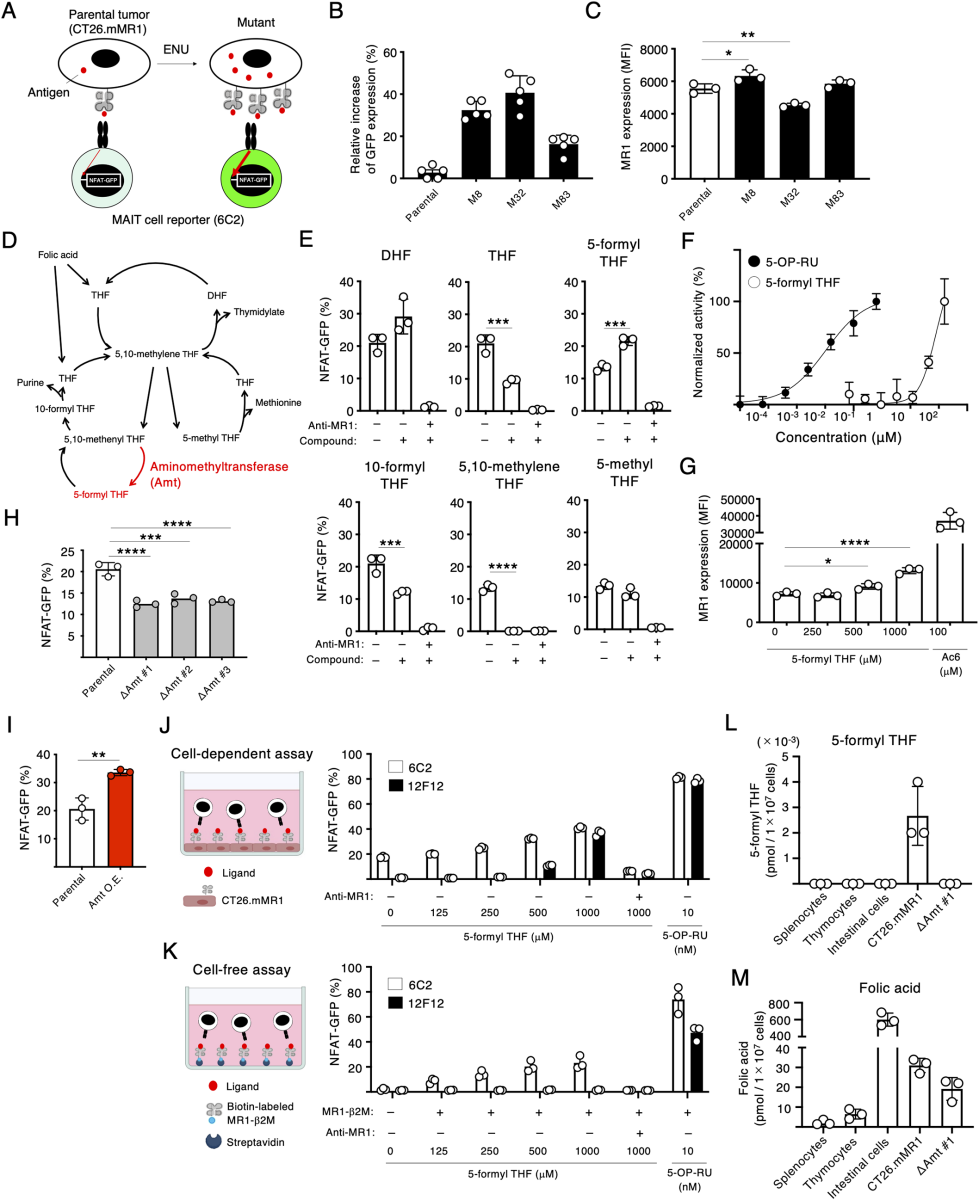
5-Formyl THF is a tumor-derived ligand for mouse MAIT cells. **(A)** Illustration of ENU-based mutagenesis screening strategy. **(B, E, F, H, I)** NFAT-GFP expressions of 6C2 cells were analyzed using flow cytometry after co-culture with CT26.mMR1 cells (parental) and their mutants (M8, M32, M83) **(B)** or CT26.mMR1 cells **(E, F)** or CT26.mMR1 cells (parental) and its *Amt*-deficient mutants (ΔAmt #1-3) **(H)** or CT26.mMR1 cells (parental) and *Amt*-overexpressing CT26.mMR1 cells (AMT O.E.) **(I)** on the 7AAD-negative TCRβ^+^ gate. **(B)** The formula of relative change of NFAT-GFP expression is Relative increase of GFP expression (%) = [(NFAT-GFP expression value of 6C2 cells after co-culture with each mutant) – (NFAT-GFP expression value of 6C2 cells after co-culture with CT26.mMR1 cells)]/(NFAT-GFP expression value of 6C2 cells after co-culture with CT26.mMR1 cells) ×100. Data are means ± SEM from five biological replicates per group. **(C, G)** Surface MR1 expression on CT26.mMR1 cells was analyzed. Data are means ± SEM from three biological replicates per group. **(G)** Indicated doses of 5-formyl THF were added during the culture. Ac-6-FP (100 μM) was used as a positive control. **(E)** NFAT-GFP expressions of MAIT cell reporter cell lines 6C2 were analyzed after stimulation with various intermediates in folate metabolism pathway (500 μM). Data are means ± SEM from three biological replicates per group. **(F)** Graph shows dose-dependent activity of 6C2 cells to 5-OP-RU and 5-formyl THF. Data are means ± SEM from three biological replicates at each time point. **(H, I)** NFAT-GFP expressions of MAIT cell reporter cell lines 6C2 were analyzed after co-culture with various antigen-presenting cells. Data are means ± SEM from three biological replicates per group. **(J, K)** NFAT-GFP expressions of MAIT cell reporter cell lines, 6C2 (filled black) and 12F12 (open), were analyzed after co-culture with CT26.mMR1 cells **(J)** or on human MR1-β_2_M-coated plates **(K)** in the presence of indicated doses of 5-formyl THF. As a control, an anti-MR1 blocking antibody (10 μg/ml) or 5-OP-RU (10 nM) was added to the culture. Data are means ± SEM from three biological replicates per group. **(L, M)** Levels of 5-formyl THF and folic acid were analyzed by LC–MS analysis. Data are means ± SEM from three biological replicates per group. **(D)** Illustration shows folate metabolism pathway which was modified from the previous study (31). **(C, E, G-I)** Statistical significance was determined by one-way ANOVA, followed by Dunnett’s multiple comparison test **(C, E, G, H)** or unpaired two-tailed Student’s *t*-test **(I)** (*p < 0.05, **p < 0.01, ***p < 0.001, ****p < 0.0001). **(A, J)** The graphics were created with BioRender.com. **(A-K)** Data are representative of two independent experiments.

Next, we sought to determine which tumor-derived ligands presented by MR1 were recognized by the mouse MAIT cells. To identify the ligands, we treated CT26.mMR1 cells with a chemical mutagen, *N*-ethyl-*N*-nitrosourea (ENU), to generate mutants with different agonistic activities toward 6C2 cells (**Figure 2A**). After screening 99 mutants (**Supplementary Figure S2A**), we identified three mutants (referred to as M8, M32, M83), which enhanced GFP expression by 6C2 cells (**Figure 2B**). Since MR1 expression is increased after MR1 captures a natural ligand (32), we searched for mutants with high MR1 expression. As MR1 expression in M8, but not the other mutants (M32 and M83), was significantly enhanced compared to expression in the parental tumor cells (**Figure 2C**), we focused on M8 to search for agonistic MAIT cell ligands. Comparative gene expression analysis revealed that 199 metabolic pathway gene expressions were augmented in M8 compared to the parental tumor CT26.mMR1 cells (Data file S1). These pathways included folate metabolism pathway-related twelve genes (**Supplementary Figure S2B**; **Data file S1**), which increase expressions in various cancers including CRCs (33). The agonistic activity of intermediates in this pathway was then tested using 6C2 cells (**Figure 2D**). Of the metabolites, THF, 10-formyl THF, and 5,10-methylene THF had an antagonistic effect on 6C2 cells, while 5-formyl THF led to dose-dependent agonistic activity (**Figures 2E, F**), with this activity being TCR-dependent, as evidenced by a lack of GFP expression by 6C2 cells when an anti-MR1 antibody was added (**Figure 2E**). Furthermore, exogenous 5-formyl THF induced the dose-dependent upregulation of MR1 (**Figure 2G**). No cytotoxic effects of 5-formyl THF were observed when using CT26.mMR1 cells (**Supplementary Figure S2C**).

Since 5-formyl THF is an intermediate in the folate metabolism pathway, we then sought to determine if other downstream metabolites converted from 5-formyl THF were mouse MAIT cell ligands. To test this, a clustered regularly interspaced short palindromic repeats (CRISPR)-Cas9 strategy was implemented to disrupt *Aminomethyltransferase* (*Amt*), thereby impacting the generation of 5-formyl THF (**Figure 2D**). We established three independent *Amt*-deficient CT26.mMR1 cell lines, with the disruption of the gene being confirmed by sequencing (**Supplementary Figure S2D**). Liquid Chromatography-Mass Spectrometry (LC–MS) analysis provided further evidence that the biosynthesis of 5-formyl THF was reduced in the *Amt*-deficient CT26.mMR1 cells (**Supplementary Figures S2E–G**). These *Amt*-deficient colorectal tumors also reduced the reporter activities of the 6C2 cells (**Figure 2H**) and their MR1 expression level (**Supplementary Figures S2H, I**), compared to parental CT26.mMR1 cells. Conversely, *Amt*-overexpressing CT26.mMR1 cells increased the reporter activity of 6C2 cells (**Figure 2I**) and MR1 expression (**Supplementary Figures S2J, K**). Exogenous 5-formyl THF to *Amt*-deficient CT26.mMR1 cells recovered the reporter activities of the 6C2 cells to the same level in the parental CT26.mMR1 cells (**Supplementary Figure S3A**). The agonistic activity after stimulation with 5-formyl THF was not limited to 6C2 cells, as evidenced by the activation of 12F12 cells by 5-formyl THF at higher (≥ 500μM) ligand concentrations (**Figure 2J**). The 5-formyl THF-dependent MAIT cell agonistic activity of both 6C2 and 12F12 cells was attenuated in the presence of Ac-6-FP (**Supplementary Figures S3B, C**). 5-OP-RU-dependent MAIT cell agonistic activity of both 6C2 and 12F12 cells was unaffected in the presence of 5-formyl THF, which indicates that 5-formyl THF binds MR1 with lower affinity than 5-OP-RU (**Supplementary Figures S3D, E**). Taken together, these data suggest that 5-formyl THF is a MAIT agonist. However, the involvement of molecules other than 5-formyl THF could not be discounted because *Amt*-deficiency in CT26.mMR1 cells led to attenuated proliferation (**Supplementary Figure S3F**), which is possibly due to global effects on other metabolic pathways (34). To investigate this, an antigen-presenting cell-free reporter assay using human MR1-beta-2-microglobulin (β_2_M) complex was conducted. Previously, it has been shown that mouse and human MAIT cells exhibit cross-reactivity to mammalian MR1 orthologues (35). We also observed that the human MR1-β_2_M complex induced GFP reporter activity of both human and mouse MAIT cell lines, 4L4T and 6C2, upon stimulation with 5-OP-RU (**Supplementary Figures S3G, H**). Using this cell-free antigen-presentation system, a dose-dependent increase in GFP reporter activity to 5-formyl THF was observed in 6C2 but not 12F12 cells (**Figure 2K**). 5-Formyl THF was detectable only in CT26.mMR1 cells but not in normal cells, despite the presence of folic acid, a starting compound of the folate metabolism pathway, in all cells analyzed in this study (**Figures 2L, M**). These results suggest that CRC-derived 5-formyl THF in CT26.mMR1 cells activates 6C2 cells via the MAIT TCR, whereas 12F12 cells recognize other newly synthesized ligands by antigen-presenting cells after incubation with 5-formyl THF.

### 2.4 The introduction of mouse MAIT TCRs confers reactivity to 5-formyl THF

**Figure 3:**
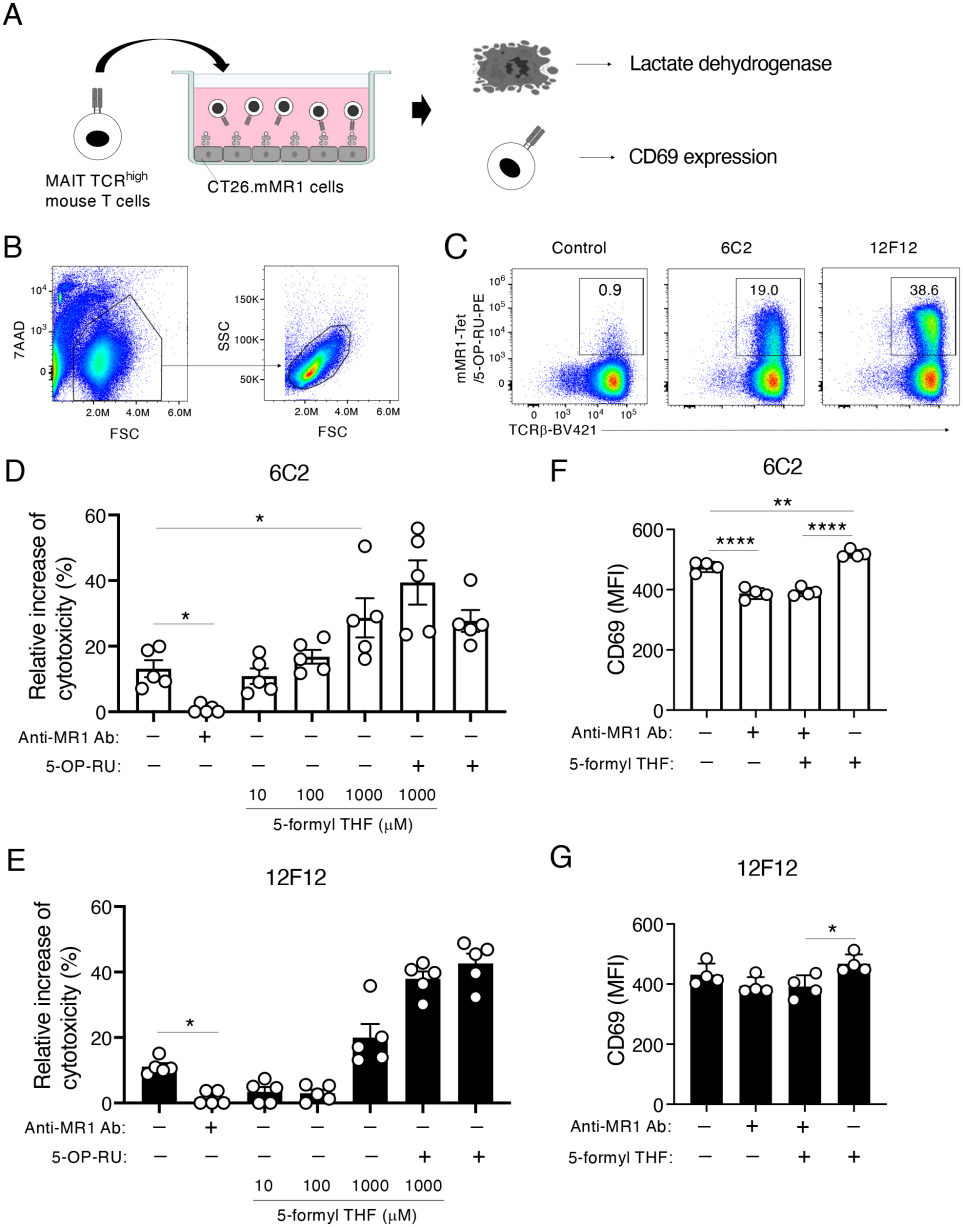
5-Formyl THF induces tumor-killing activity through mouse MAIT TCRs. **(A)** Schematic representation of tumor killing assay. The graphics were created with BioRender.com. **(B)** Gating strategies for flow cytometric analysis. After excluding 7AAD^+^ dead cells, lymphocytes were identified based on parameters of forward scatter (FSC) and side scatter (SSC). **(C)** After gating on **(B)**, cells were further plotted using anti-TCRβ antibody and 5-OP-RU-loaded mouse MR1 tetramer. **(D, E)** After MAIT TCR-overexpressing mouse T cells from BALB/c-background *Traj33*^–/–^ mice were co-cultured with CT26.mMR1 cells, cytotoxic activity was analyzed by LDH release. Data are means ± SEM from five biological replicates per group. An anti-MR1 blocking antibody (25 μg/ml) or 5-formyl THF or 5-OP-RU (10μM) was added during the co-culture. Relative increase of % cytotoxicity to anti-MR1-treated groups was shown. **(F, G)** After the co-culture as in **(D, E)**, CD69 expressions on MAIT TCR-overexpressing mouse T cells were analyzed by flow cytometry after gating on 5-OP-RU-loaded mouse MR1 tetramer^+^ cells. Bar graphs show mean fluorescence intensity (MFI) values of CD69 expression. Data are means ± SEM from four biological replicates per group. **(D–G)** Statistical significance was determined by one-way ANOVA, followed by Dunnett’s multiple comparison test (*p < 0.05, **p < 0.01, ****p < 0.0001). Data are representative of two independent experiments.

To assess the functional activity of mouse MAIT TCRs in primary T cells and to determine whether it was enhanced by 5-formyl THF, mouse MAIT TCRs were retrovirally transduced to T cells derived from MAIT cell-deficient *Traj33*^–/–^ mice. Cytotoxic activity against CT26.mMR1 cells was then examined as a readout for TCR-dependent activity (**Figure 3A**). After MAIT TCR introduction, the surface expression of the MAIT TCR was confirmed by staining with mouse 5-OP-RU-loaded MR1 tetramer (mMR1-Tet) (**Figures 3B, C**). Following the co-culture of the mMR1-Tet^+^ cells with the mouse CT26.mMR1 cells, the tumor killing activity of the 6C2 and 12F12 mouse MAIT TCR-overexpressing T cells, which was determined by the release of the apoptotic marker, lactate dehydrogenase (LDH), was found to be reduced in the presence of anti-MR1 antibody (**Figures 3D, E**). In addition, the cytotoxic activity induced by 5-formyl THF increased in a dose-dependent manner (**Figures 3D, E**). As the 6C2 and 12F12 mouse MAIT TCR-overexpressing T cells also expressed endogenous MR1, fratricide effect, rather than tumor killing activity, could contribute to increased LDH release upon stimulation with 5-formyl THF (**Supplementary Figure S4A**). However, this was unlikely because after stimulation with 5-formyl THF, the number of 6C2 and 12F12 mouse MAIT TCR-overexpressing T cells was not changed in the presence of an anti-MR1 blocking antibody (**Supplementary Figure S4B**). In support of the TCR-dependent tumor killing activity, it was also observed that CD69 expression (a marker for TCR-mediated signaling) decreased on mouse MAIT TCR-overexpressing T cells when the anti-MR1 antibody was added to cells stimulated with 5-formyl THF (**Figures 3F, G**). Taken together, these results suggest that mouse MAIT TCRs are responsible for the agonistic activity that is observed in the presence of 5-formyl THF.

### 2.5 5-formyl THF–reactive MAIT cells are present in humans

**Figure 4:**
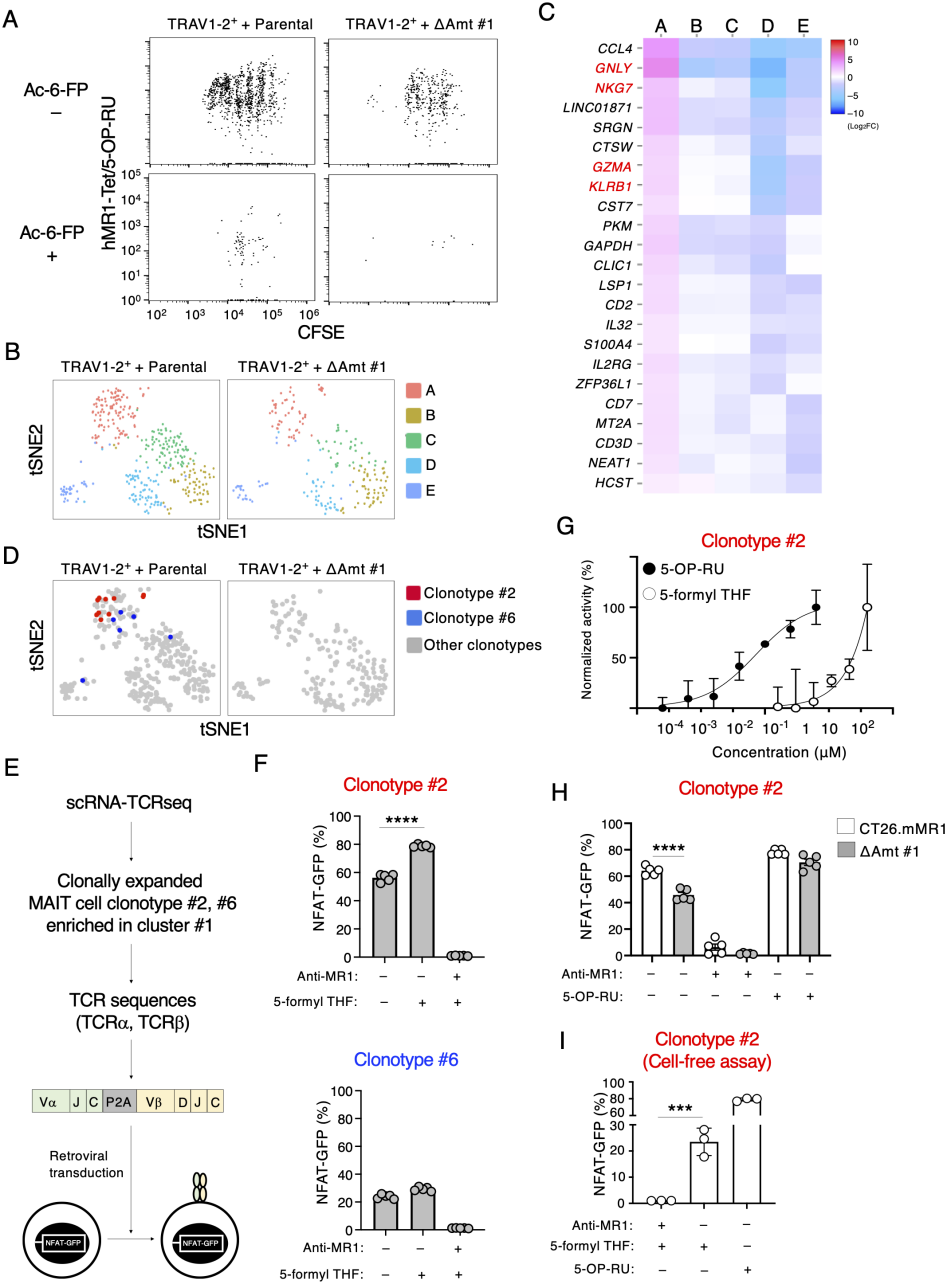
Identification of 5-formyl THF-reactive human MAIT cells by single cell analysis. **(A)** After enrichment of TRAV1-2^+^ cells from PBMCs, cells were labeled with CFSE. The CFSE-labeled TRAV1-2^+^ cells were co-cultured with mitomycin C-treated *Amt*-sufficient (parental) or *Amt*-deficient (ΔAmt #1) CT26.mMR1 cells for seven days. Plots show a representative from three experiments (See **Supplementary Figure S5B**). Dot plots show 7AAD-negative cells after the co-culture in the presence or absence of Ac-6-FP (100μM). **(B)** t-SNE plots of *TRAV1-2*^+^ cells generated by data based on single cell transcriptome analysis. Colored clusters were identified using Loupe Cell Browser provided by 10x Genomics (https://www.10xgenomics.com/support/software/loupe-browser/downloads). **(C)** Heatmap analysis of five clusters identified in **(B)** with log_2_ fold-change (FC). **(D)** Clonotypes identified by single cell TCR analysis; clonotype #2 (red), clonotype #6 (blue) and other clonotypes (grey). **(E)** Schematic representation for establishment of MAIT reporter cells based on scRNA-TCR-sequencing analysis. **(F, H)** Bar graphs show NFAT-GFP expressions of reporter cell lines expressing clonotype #2 and #6 TCRs, after co-culture with CT26.mMR1 cells and its *Amt*-deficient mutant cells (ΔAmt #1) in the presence or absence of an anti-MR1 antibody (10μg/ml) or 5-OP-RU (10nM) or 5-formyl THF (1000μM). Data are means ± SEM from five biological replicates per group. **(I)** Clonotype #2 was cultured on human MR1-β_2_M-coated plates in the presence of 5-formyl THF (1000 μM). As a control, an anti-MR1 blocking antibody (10 μg/ml) or 5-OP-RU (10 nM) was added in the culture. Data are means ± SEM from three biological replicates per group. **(F, H, I)** Statistical significance was determined by one-way ANOVA, followed by Dunnett’s multiple comparison test **(F, I)** or unpaired two-tailed Student’s *t*-test **(H)** (*p < 0.01, ****p < 0.0001). **(G)** Graph shows dose-dependent activity of clonotype #2 to 5-OP-RU and 5-formyl THF after co-culture with CT26.mMR1 cells. Data are means ± SEM from three biological replicates at each time point. **(F–I)** Data are representative of two independent experiments.

To identify 5-formyl THF-responsive human MAIT cell clonotypes, we co-cultured magnetic bead-enriched TRAV1-2^+^ human T cells with *Amt*-sufficient (parental) or *Amt*-deficient (ΔAmt #1) mouse CT26.mMR1 cells for seven days. After co-culture, human MAIT cells, which were identified by 5-OP-RU-loaded human MR1 tetramers, proliferated more than those with *Amt*-deficient tumors (upper two panels in **Figure 4A**; **Supplementary Figures S5A, B**). This proliferation was markedly reduced via the addition of the MAIT cell antagonist Ac-6-FP (lower two panels in **Figure 4A**), suggesting TCR-dependent proliferation, with a limited contribution from TCR-independent signals such as IL-12 and IL-18 (36, 37). To identify 5-formyl THF-reactive clonotypes, the cultured cells were purified by fluorescence-activated cell sorting to remove dead cells. Viable cells were subjected to single-cell RNA-TCR-sequencing analysis. We retrieved 2,392 and 1,675 cells that were co-cultured with *Amt*-sufficient (parental) and *Amt*-deficient (ΔAmt #1) mouse CT26.mMR1 cells, respectively, and the median numbers of detected genes were 576 and 508, respectively. In these samples, *TRAV1–2* expression was detected in 374 and 209 cells (**Supplementary Figure S5C**). After extracting the *TRAV1-2*^+^ populations from all cells, dimension reduction analysis using the t-Distributed Stochastic Neighbor Embedding (t-SNE) approach showed that the *TRAV1-2*^+^ cells were partitioned into five clusters A to E (**Figure 4B**). Among these, cells in cluster A expressed higher levels of genes such as *GNLY, NKG7, GZMA*, and *KLRB1* (**Figure 4C**), which are reportedly induced by human MAIT cells following TCR-dependent activation (38–41). Using TCR repertoire analysis, seventeen clonotypes that share identical nucleotide sequences for both the TCRα and TCRβ chains were identified (Data file S2). After projection of the TCR data to the t-SNE plot, more than four dots corresponding to clonotypes #2 and #6 were detected in the presence of *Amt*, but not in the absence of *Amt*, and these clonotypes were highly enriched in cluster A (**Figures 4B-D**; **Supplementary Figure S5D**). This suggests clonal expansion, which is a hallmark of antigen recognition. However, there remained a possibility that these clonotypes were present before being cultured with tumors and that they were not generated by clonal expansion. Another possibility was that soluble factors, which were produced after the human T cells were co-cultured with mouse tumor cells, induced human MAIT cell expansion in a TCR-independent manner.

To formally test the TCR-dependent responsiveness of clonotypes #2 and #6 to 5-formyl THF, GFP reporter cell lines expressing these TCRs were generated (**Figure 4E**). The agonistic activity of the reporter cell line expressing clonotype #2 TCR, but not clonotype #6 TCR, was increased via the addition of 5-formyl THF (**Figure 4F**) in a dose-dependent manner (**Figure 4G**). In support of this 5-formyl THF-dependent activity, it was also noted that GFP expression of the reporter cell line expressing clonotype #2 TCR was decreased in the absence of *Amt* (**Figure 4H**). This 5-formyl THF-dependent activity was also confirmed using an antigen-presenting cell-free assay (**Figure 4I**).

Using a Cell Trace Violet (CTV) dilution assay, it was also determined that, like the weak agonistic MAIT cell ligand, cholic acid 7-sulfate (CA7S) (41), 5-formyl THF did not induce strong TCR-dependent proliferation while 5-OP-RU led to TCR-dependent proliferation (**Supplementary Figure S5E**). The reporter cell line expressing clonotype #2 TCR was activated upon stimulation with 5-OP-RU (**Figures 4G, H**) and was positive for 5-OP-RU-loaded human MR1 tetramer, although the mean fluorescence intensity (MFI) of the 5-OP-RU-loaded human MR1 tetramer was lower than that of a typical human MAIT cell line 4L4T (**Supplementary Figure S5F**) (42). This result supports the previous study where different TCRβ usage was found to contribute to the recognition of the 5-OP-RU-MR1 complex (43). In other work, MAIT cells could be detected with a 6-FP-loaded human MR1 tetramer (21). However, we did not observe such tetramer binding to the 5-formyl THF-reactive MAIT cell line (**Supplementary Figure S5F**).

**Figure 5:**
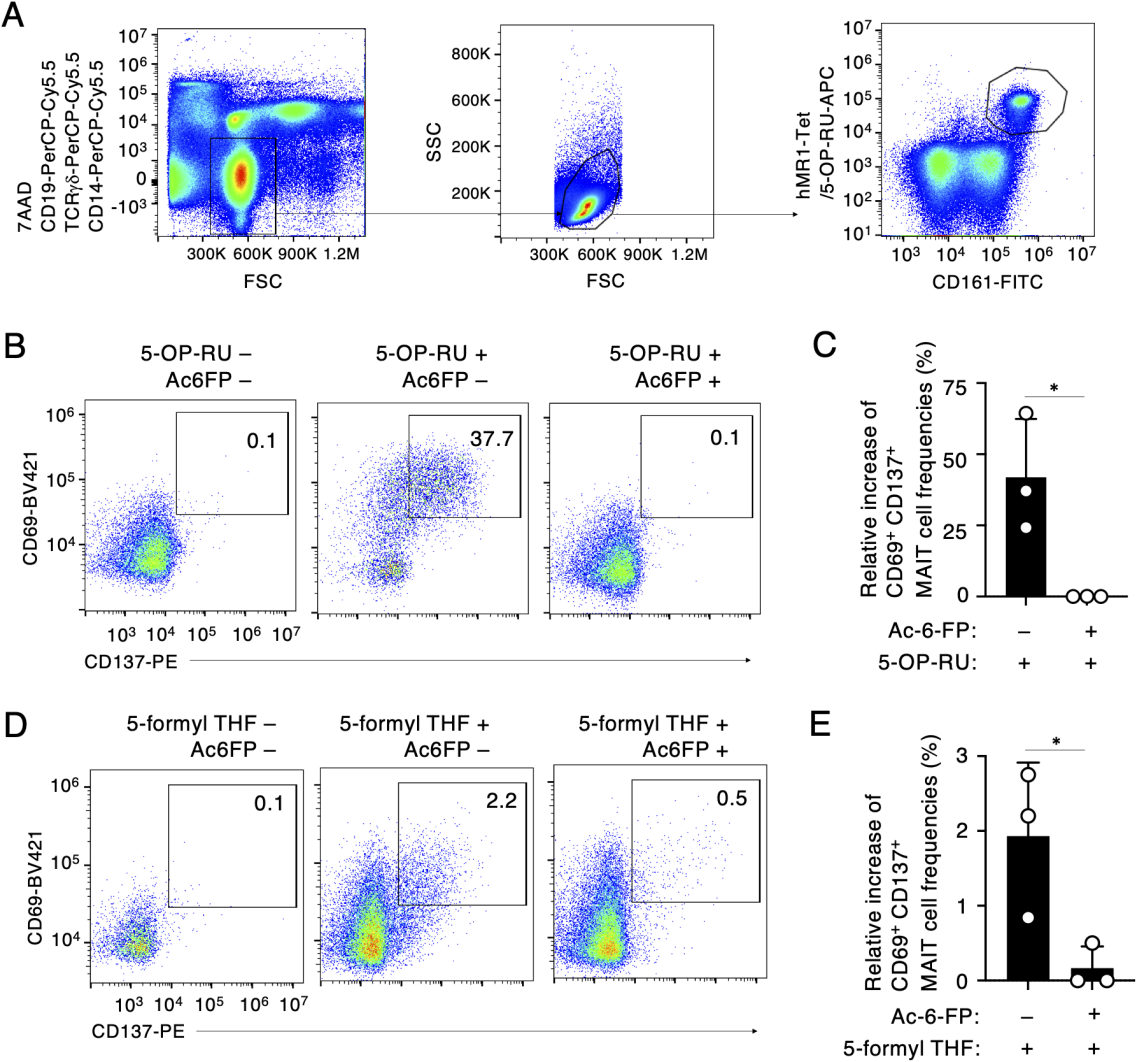
5-Formyl THF-reactive MAIT cells are present in humans. **(A)** Gating strategy of flow cytometric analysis for human PBMCs. After excluding 7AAD^+^ dead cells and non-αβ T cell populations positive for CD19, γδTCR and CD14, human MAIT cells were identified by a 5-OP-RU-loaded human MR1 tetramer and an anti-CD161 antibody. **(B–E)** After stimulation of human PBMCs with 5-OP-RU (10nM) **(B, C)** and 5-formyl THF (1000μM) **(D, E)** in the presence or absence of Ac-6-FP (500μM) for 24hr, CD137 and CD69 expression on MAIT cells was analyzed by flow cytometry. **(B, D)** Plots show CD137 and CD69 expression after gating on MAIT cells. Numbers in the plots indicate the percentages of CD137^+^ CD69^+^ MAIT cells. **(C, E)** Bar graph shows relative increase of CD137^+^ CD69^+^ MAIT cell frequencies to no-treatment control from three healthy donors. Data are means ± SEM from three biological replicates per group. Statistical significance was determined by unpaired two-tailed Student’s *t*-test (*p < 0.05, **p < 0.01). Data are representative of two independent experiments.

**Figure 6:**
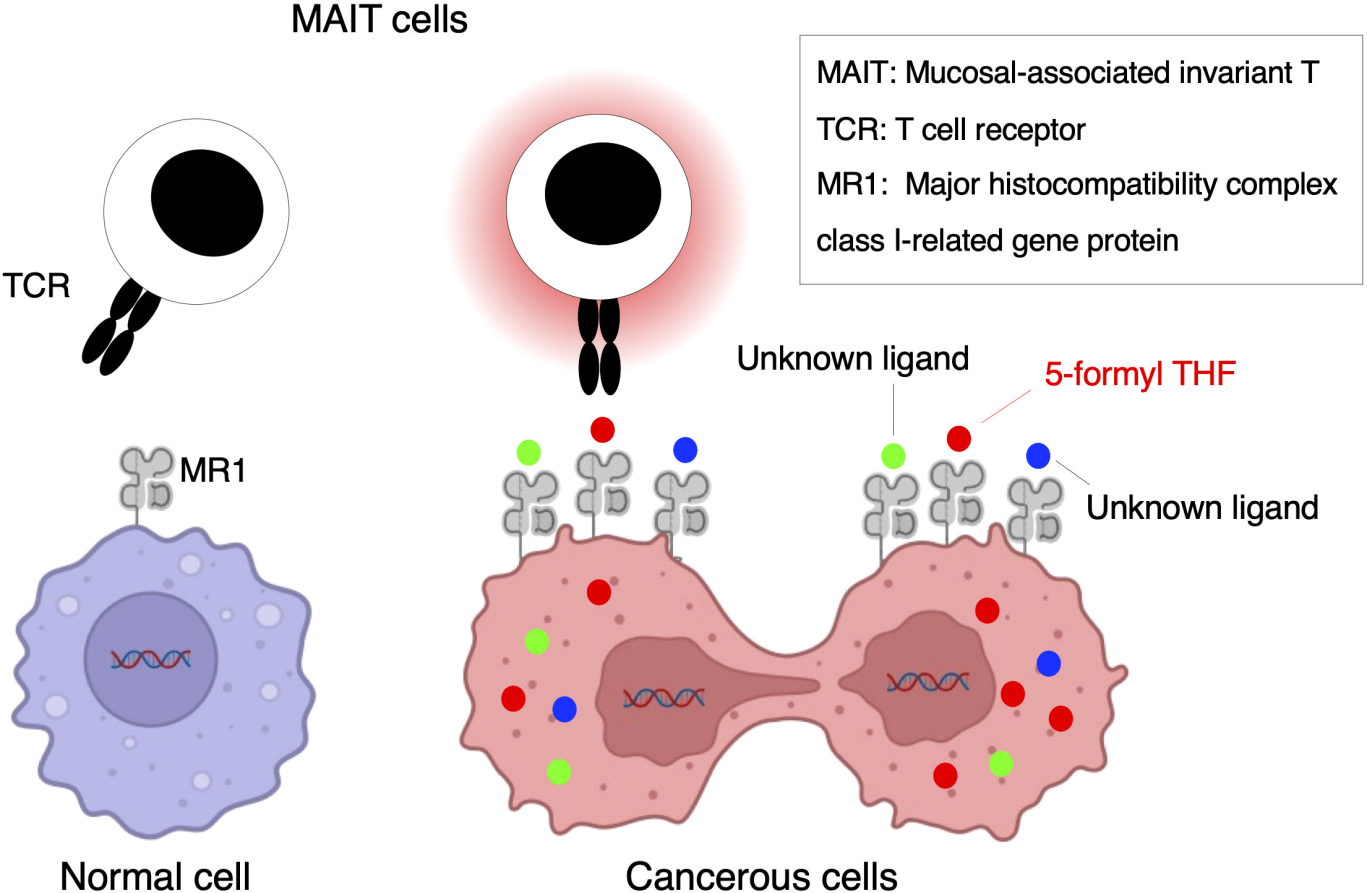
Proposed model illustrating self and neo-self-discrimination by MAIT cells. The illustration depicts that MAIT cells recognize 5-formyl THF produced by cancerous cells rather than by normal cells. The graphics were created with BioRender.com (https://www.biorender.com).

We next analyzed 5-formyl THF-reactive MAIT cell frequencies in human peripheral blood mononuclear cells (PBMCs). Reactivity was assessed based on CD69 and CD137 expression, which was transiently induced on both MAIT cells and non-canonical MR1-restricted αβ T cells following TCR-dependent activation (20, 21). Human MAIT cells were identified using a 5-OP-RU-loaded human MR1 tetramer and an anti-CD161 antibody (**Figure 5A**). Stimulation with 5-OP-RU markedly increased the frequency of CD69^+^CD137^+^ MAIT cells, whereas 5-formyl-THF induced only a modest but reproducible increase (**Figures 5B–E**). This response was abrogated by Ac-6-FP, indicating MR1-dependent activation. Consistent with these findings, 5-formyl-THF exhibited substantially weaker agonistic activity than 5-OP-RU (**Figures 5B–E**), as also observed in mouse MAIT cells (**Figure 2F**). Taken together, these data suggest that, like mouse MAIT cells, 5-formyl THF can be recognized as a weak, MR1-dependent ligand for human MAIT cells (**Figure 6**).

## 3 Discussion

In light of the recent successes of immunotherapy using immune checkpoint inhibitors, there has been much interest in the identification and function of T cells that recognize tumor-derived ligands. Accumulating evidence has revealed that diverse T cell populations which infiltrate tumor sites recognize various ligands, including peptides and metabolites (4, 44). Accordingly, the identification of agonistic tumor-derived T cell ligands that potentially induce anti-tumor activity will help establish an alternative and complementary approach for cancer immunotherapy. In the present study, we identified 5-formyl THF, which is generated during DNA synthesis pathway, as the first tumor-derived MAIT cell ligand (**Figure 6**); however, its functional relevance *in vivo* remains to be determined.

Apart from 5-formyl THF, no other tumor-derived MAIT cell ligands have been identified, although some non-MAIT tumor-reactive MR1-restricted αβ T cells and their antigens have been reported (5, 20, 45). This raises the possibility that 5-formyl THF may be recognized by non-MAIT MR1-restricted αβ T cells, but we reason that this is unlikely. The previously described non-MAIT MR1-restricted αβ T cells do not recognize the microbial MAIT cell agonist 5-OP-RU and thus, have a different binding mode compared to the MAIT cells in our study. Our work also points to the presence of other tumor-derived MAIT cell ligands, as illustrated by the observed human and mouse MAIT cell reporter activity when these cells were co-cultured with *Amt*-deficient tumor cells that lack 5-formyl THF (**Figures 2H**, **4H**). Other evidence for the presence of alternative tumor-derived MAIT cell ligands comes from the unique agonistic activity of 12F12 cells, which do not recognize 5-formyl THF but are activated in a TCR-dependent fashion following co-culture with tumor cells grown in 5-formyl THF-rich media (**Figures 2J, K**).

A previous study (26) showed that the E96A mutation, but not the F97A mutation, in the CDR3β loop abrogated IL-2 production by 6C2 cells following co-culture with the mouse embryonic fibroblast LM1.8 cells overexpressing mouse MR1. In our work, when the 6C2 reporter cell line was co-cultured with NIH3T3.mMR1 cells and CT26.mMR1 cells, we observed a similar reduction in MAIT cell activation. For example, the E96A mutant led to a greater decrease in GFP reporter cell activity, whereas the F97A mutant had a comparatively small reduction in GFP expression. These results suggest that 5-formyl THF is also produced by the mouse embryonic fibroblast LM1.8 cell line.

The physiological functions of 5-formyl THF are not well understood. Although 5-formyl THF was undetectable in normal cells in this study, it is generally considered to be synthesized by all living cells, rather than being specific to cancerous cells. Most tetrahydrofolate species play an indispensable role in the broad set of biochemical transformations known as one-carbon metabolism, whereas 5-formyl THF is not thought to be essential for this pathway (31). It might be that 5-formyl THF plays a role in the thymic selection of MAIT cells that undergo selection by double-positive cells. T cell subsets, including MAIT cells, develop in the thymus upon recognition of selecting antigens, with commensal-derived antigens 5-OP-RU and CA7S being suggested to play a role in this process (18, 41). Because germ-free mice which lack 5-OP-RU and CA7S still exhibit a small, but substantial, number of thymic MAIT cells (41, 46), there is the possibility that other unknown molecules, such as 5-formyl THF, are involved in thymic MAIT cell selection. Genetic inactivation of *Amt* causes fetal death in mice (47), however, conditional ablation of *Amt* may allow us to clarify the role of Amt in the thymic selection of MAIT cells. In any case, we observe 5-formyl THF in tumor cells but not healthy cells. This suggests that tumor cells produce more of the MAIT cell ligand, possibly as a result of their persistently high levels of DNA synthesis compared with normal cells. Amt-dependent genes identified in our study will provide insight into the functional roles of Amt in cancer cells. Notably, higher *AMT* expression has been associated with improved survival in colorectal cancer based on publicly available datasets (Human Protein Atlas, https://www.proteinatlas.org/), raising the possibility that AMT-dependent production of MAIT cell ligands such as 5-formyl THF may enhance tumor immune surveillance. In this context, tumor models using Amt-deficient cancer cells will be particularly valuable to directly assess whether loss of Amt alters MAIT cell activation and contributes to tumor immune evasion *in vivo*. Alternatively, given that 5-formyl-THF induced relatively modest MAIT cell activation, this lower potency may reflect a distinct physiological role, in which weak MR1 ligands contribute to the fine-tuning of MAIT cell activity and maintenance of immune homeostasis, rather than driving strong effector responses as reported for the weak MAIT cell ligand CA7S (41).

It should also be noted that, clinically, 5-formyl THF is called leucovorin, and as such, has been used in combination with fluorouracil (5-FU) for CRC patients undergoing chemotherapy (31). Leucovorin acts synergistically with 5-FU to block the activity of thymidylate synthase, which is essential for DNA replication (48–50), and thus leads to enhanced anti-tumor effects during chemotherapy (51, 52). Considering the data from our studies, it might also be possible that the synergistic activity of 5-FU and 5-formyl THF may be partially mediated by MAIT cell activity.

Our data show the ability of 5-formyl THF to induce TCR-dependent tumor-killing activity by MAIT cells *in vitro*. However, the tumor-killing activity observed in this study may not fully recapitulate physiological conditions *in vivo* as our experimental system uses MR1-overexpressing cells and a high dose of 5-formyl THF. Notwithstanding, MAIT cell activity is modulated by TCR-independent factors, including costimulatory molecules (CD2, CD28 and ICOS) (53, 54), an inhibitory molecule PD1 (55), and cytokines (IL-18 and IL-12) (36, 37). Such complex regulatory mechanisms may explain, at least in part, why TCR-dependent MAIT cell activation by a strong agonist 5-OP-RU induces both tumor-promoting and tumor-suppressive activity *in vivo* (9, 10). In this context, relatively low-potency MR1 ligands—including diclofenac metabolites, riboflavin derivatives, JYM72, CA7S and 5-formyl THF—may contribute to the fine-tuning of MAIT cell activity (41, 56–58). Furthermore, emerging evidence indicates that ligand stability and persistence are key determinants of MAIT cell activation (58–60), suggesting that even weak ligands may exert biological effects. Notably, JYM72 induced comparable anti-tumor effect to 5-OP-RU (11). Further *in vivo* studies will be required to determine the anti-cancer potential of 5-formyl THF, and perhaps other tumor-derived MAIT cell ligands, in the cancer microenvironment. It would also be instructive to determine if 5-formyl THF plays a role in other immunological processes, including thymic MAIT cell selection. Investigations such as these will shed valuable light on the complex roles that MAIT cells have in health and disease.

## 4 Materials and methods

### 4.1 Mice

BALB/c mice (Kyudo, Japan; RRID: MGI:2161072) were purchased from Kyudo. *Traj33*^–/–^ mice were generated as described previously (61). *Traj33*^–/–^ mice were backcrossed to BALB/c mice more than ten generations. 7-week-old female mice were used in this study. This study was approved by the Committee of Ethics on Animal Experiments in the Faculty of Medicine, Yamaguchi University (Approved number: J18015) and Kyushu University (Approved number: A25-084). Experiments were carried out under the control of the Guidelines for Animal Experiments BALB/c mice were purchased from Kyudo. *Traj33*^–/–^ mice were generated as described previously (61). *Traj33*^–/–^ mice were backcrossed to BALB/c mice more than ten generations. 7-week-old female mice were used in this study. This study was approved by the Committee of Ethics on Animal Experiments in the Faculty of Medicine, Yamaguchi University (Approved number: J18015) and Kyushu University (Approved number: A25-084). Experiments were carried out under the control of the Guidelines for Animal Experiments.

### 4.2 Compounds

Ac-6-FP was purchased from Schircks Laboratories. Methylglyoxal (Cat. #M0250), Dihydrofolic acid (DHF) (Cat. #4033-27-6) and 5,6,7,8-tetrahydrofolate (THF) (Cat. #135-16-0) were purchased from Sigma-Aldrich. 5-formyl THF (Cat. #FC61510) was purchased from Biosynth Ltd. 10-Formyl-5,6,7,8-tetrahydro folic acid (10-formyl THF) (Cat. #F701095), 5,10-Methylenetetrahydrofolate (5,10-methylene THF) (Cat. #3432-99-5), 5-Methyltetrahydrofolic acid (5-methyl THF) (Cat. #134-35-0) and 5-Amino-6-(d-ribitylamino) uracil (5-A-RU) (Cat. #A629245) were purchased from Toronto Research Chemicals. 5-OP-RU was generated by reacting 5-A-RU in DMSO with an equal molar ratio of methylglyoxal. 5-OP-RU concentrations are shown under the assumption that all 5-A-RU is converted to 5-OP-RU.

### 4.3 Cell lines used in this study

Human and mouse MR1-overexpressing antigen-presenting cells were generated by retroviral gene transduction of mouse MR1 into mouse fibroblastic cell line NIH3T3 (RRID: CVCL_0594) and mouse colorectal tumor cell line CT26 (RRID: CVCL_7254) (NIH3T3.mMR1 and CT26.mMR1 cells). CT26 cells lacking *Amt* (ΔAmt #1-3) were generated by CRISPR-Cas9 system. Off-target analyses were performed using CRISPR direct software (https://crispr.dbcls.jp) and CCTop CRISPR/Cas9 target online predictor (https://cctop.cos.uni-heidelberg.de). Targeted sequences for disrupting the *Amt* genes were shown as follows: CCCTCTGGGTTTTCGCCTGCAGG, GAGTGGGCGACTCTGGACCAAGG and GCCTGCAGGCGAAAACCCAGAGG. These sequences were cloned into the pX330.puro vector (Addgene). pX330.puro vector was a gift from Sandra Martha Gomes Dias (Addgene plasmid #110403; http://n2t.net/addgene:110403; RRID: Addgene_110403). After cloning into pGreenPuro expression vector (System Biosciences), *Amt* gene was lentivirally transduced to the CT26.mMR1 cells. *Amt*-overexpressing CT26.mMR1 cells were selected by puromycin. For generating mutants (M8, M32, M83), ENU (0.3 mg/mL)-treated CT26.mMR1 tumors were cloned by limiting dilution and each clone was screened by flow cytometry based on surface MR1 expression.

### 4.4 Reporter assay

Human and mouse MAIT αβTCR-expressing reporter cell lines (TCRs were derived from 6C2, 12F12, 4L4T, clonotype #2 and clonotype #6) were generated by retroviral gene transduction of MAIT αβTCRs into the mouse TCR-negative thymoma (23, 62). Full TCRα and TCRβ sequences for clonotype #2 and clonotype #6 are provided upon request. Mouse MAIT αβTCR sequences (6C2, 12F12) were provided by Prof. Olivier Lantz (Institute Curie, France). For cell-mediated antigen presentation assay, antigen-presenting cells (1 × 10^4^ cells per well in 96-well plates) were cultured overnight at 37 ˚C in a CO_2_ incubator. For cell-free antigen-presentation assay, human MR1-β_2_M complex (100ng per well, ACRO Biosystems) was incubated in streptavidin-coated 96-well plates (TOMSIC) at 4˚C overnight. Next day, the plates with antigen-presenting cells or human MR1-β_2_M-coated plates were treated with 100 μM of Ac-6-FP or 10 μg/mL of anti-MR1 antibody as a negative control for 30 minutes at 37˚C. After the incubation, agonistic ligands such as 5-formyl THF and 5-OP-RU (10nM) and MAIT αβTCR-expressing reporter cell lines (5 × 10^4^ cells per well in 96-well plates) were added in the culture. After 24 hours of co-culture, NFAT-GFP expressions in reporter cell lines were analyzed by flow cytometry after gating on 7-Amino-Actinomycin D (7AAD)-negative TCRβ^+^ cells. Positivity of NFAT-GFP expression was set at 1% in the group of Ac-6-FP-treated or anti-MR1-treated cells. The same gating was applied for other samples.

### 4.5 Flow cytometry

For MR1 staining on tumor cells, PE-labeled anti-MR1 antibody [clone 26.5; BioLegend, Cat# 361106, RRID: AB_2563043] or its isotype control [BioLegend, Cat# 400907, RRID: AB_326593] was added at 10 μg/mL for the last 4 hours of the culture as previously described (63). For proliferation assays, cells were stained with 1 μM of Carboxyfluorescein succinimidyl ester (CFSE) or CTV for 20 minutes at 37 ˚C in a CO_2_ incubator. For surface staining of mouse lymphocytes and human PBMCs, single-cell suspensions were stained for 30 min on ice with antibodies as listed in Data file S3. For tetramer staining, prior to cell surface staining, cells were stained with 5-OP-RU-loaded human or mouse MR1 tetramers (NIH Tetramer Core Facility) or an empty human MR1 tetramer (MBL) loaded with 5-OP-RU or 6-formyl pterin (6-FP) for 30 min at room temperature. After cell surface staining, dead cells were detected with a 7AAD-containing viability staining solution (BioLegend).

### 4.6 RNA sequencing

Total RNA from CT26.mMR1 cells and their mutants was extracted by RNA Basic Kit (FastGene). Library preparation was performed using a TruSeq stranded mRNA Library Prep Kit (Illumina) according to the manufacturer’s instructions. Sequencing was performed on an Illumina NovaSeq 6000 sequencer (Illumina) in 101-base single-read mode. Sequenced reads were mapped to the mouse reference genome sequences (mm10) using TopHat version 2.2.1 (RRID: SCR_013035). The fragments per kilobase of exon per million mapped fragments (FPKMs) were calculated using Cufflinks version 2.2.1 (RRID: SCR_014597). The raw RNA-seq data were processed utilizing the online analysis platform IDEP2.0 (Integrated Differential Expression and Pathway Analysis) (http://bioinformatics.sdstate.edu/idep/) (64)(RRID: SCR_027373). After uploading read count data, differential expression analysis was performed, employing criteria of fold change (FC) > 1 and false discovery rate (FDR) < 0.1.

### 4.7 Human subjects

The study design and methods were approved by the Institutional Review Boards of the Centers for Clinical and Translational Research of Yamaguchi University Hospital (IRB number: H27–183) and Kyushu University Hospital (IRB number: 21159). Mononuclear cells from blood samples of healthy donors (Data file S4) were prepared using BD Vacutainer CPT following manufacturer’s instructions. Mononuclear cells were suspended in Cell Banker (Nihon Zenyaku Kogyo) and stored at −80 °C until use. For single-cell analysis, TRAV1-2^+^ human T cells were enriched by MojoSort Magnetic Cell Separation Kit (BioLegend) before co-culture with *Amt*-sufficient (parental) or *Amt*-deficient (ΔAmt #1) mouse CT26.mMR1 cells. The purity of TRAV1-2^+^ human T cells was more than 90%. An anti-TRAV1-2-specific antibody was used for pre-enrichment due to the low frequency of MAIT cells; however, as TRAV1–2 expression is not entirely specific, MR1 tetramer staining was subsequently used to ensure antigen specificity.

### 4.8 Single cell-based transcriptome and TCR analysis

Libraries for human T cells were prepared using following reagents: Chromium Single Cell 5’ Library & Gel Bead Kit, PN-1000120; Chromium Single Cell V(D)J Enrichment Kit, Human T Cell, PN-1000005; Chromium Single Cell 5’ Library Construction Kit, PN-1000020; Single Index Kit T Set A, PN-1000213; Chromium Single Cell 5’ Feature Barcode Library Kit, PN-1000080; and Single Index Kit N Set A, PN-1000212. CFSE-labeled TRAV1-2^+^ cells from PBMCs were co-cultured with *Amt*-sufficient (parental) or *Amt*-deficient (ΔAmt #1) CT26.mMR1 cells treated with 10 μg/mL of mitomycin C (Nakarai). After the culture, 7AAD-negative CD3^+^ cells were sorted by FACSAria cell sorter (BD Biosciences). Approximately 5 × 10^3^ cells were loaded into Chromium microfluidic chips to generate single-cell gel-bead emulsions using the Chromium controller (10x Genomics) according to the manufacturer’s recommendations. RNA from each sample was subsequently reverse-transcribed using a Veriti Thermal Cycler (Thermo Fisher Scientific), and all subsequent steps to generate single-cell libraries were performed according to the manufacturer’s protocol, with 14 cycles used for cDNA amplification. Then ∼50 ng of cDNA was used for gene expression library amplification by 14 cycles in parallel with cDNA enrichment and library construction for T cell libraries. Fragment sizes of the libraries were confirmed with the Agilent 2100 Bioanalyzer (Agilent). Libraries were sequenced on an Illumina NovaSeq 6000 as paired-end mode (read1: 28bp; read2: 91bp). The raw reads were processed by Cell Ranger 3.1.0 (10x Genomics) (RRID: SCR_023221). Using the human genome reference allows us to exclude mouse-derived antigen-presenting cells such as *Amt*-sufficient (parental) or *Amt*-deficient (ΔAmt #1) mouse CT26.mMR1 cells from the dataset. Analysis was done using Loupe Cell Browser provided by 10x Genomics (https://www.10xgenomics.com/support/software/loupe-browser/downloads) (RRID: SCR_018555). Cells with a mitochondrial content >2% and cells with <200 or >3800 genes detected were considered as outliers (dying cells and empty droplets and doublets, respectively) to be filtered out.

### 4.9 Mass spectrometry analysis

LC–MS-grade water, acetonitrile, and methanol were purchased from Kanto Chemical Co. Inc. (Tokyo, Japan). LC–MS-grade formic acid (99%) was purchased from Fujifilm Wako Pure Chemical (Osaka, Japan). Sodium ascorbate (≥98.0%) and lidocaine (≥99.0%) were purchased from Nacalai Tesque (Kyoto, Japan). Folate species extraction was performed according to previously described method (65–67). In brief, after preparing single cell suspensions of the spleen, thymus and intestine from BALB/c mice (68) or mouse tumors, cells were resuspended in either 250 μL or 1 mL of 80% methanol containing 12.5 mM sodium ascorbate. The samples were transferred to microfuge tubes, and vortexed vigorously for 1 min, sonicated for 5 min, and then centrifuged at 16,000 ×g for 5 min at 4 °C. Supernatant aliquots (200 μL or 800 μL) were transferred to 1.5 mL Eppendorf tubes, dried under a nitrogen stream, and reconstituted in 30 μL of water containing lidocaine (50 pmol) as an internal standard.

For **Supplementary Figures S2E-G**, chromatographic separation was performed on a Hypersil GOLD C18 column (1.9 µm, 2.1 × 150 mm; Thermo Fisher Scientific) with a Vanquish UHPLC System (Thermo Fisher Scientific, Waltham, MA). A 20 μL aliquot of the sample was injected into the column and eluted at a flow rate of 0.2 mL/min under the following solvent conditions: solvent A, 20 mM ammonium acetate (pH 5.4) in H_2_O; solvent B, 20 mM ammonium acetate (pH 5.4) in H_2_O: acetonitrile (80:20, v/v). The gradient program was as follows: 0–10 min of a linear gradient from 0% to 50% B; 10–11 min of a linear gradient from 50% to 100% B; 11–15 min isocratic elution of 100% B; 15–15.1 min of a linear gradient from 100% to 0% B; and finally 15.1–20 min isocratic elution of 0% B. Mass spectra were acquired using an Orbitrap Exploris 120 mass spectrometer (Thermo Fisher Scientific) equipped with an heated electrospray ionization source, with acquisition in negative ion mode. The source parameters were as follows: spray voltage of 2.5 kV, ion transfer tube temperature of 320 °C, vaporizer temperature of 275 °C, sheath gas flow of 35 units, auxiliary gas flow of 7 units, and sweep gas of 0 units. A full scan was performed in the mass-to-charge ratio (*m*/*z*) range of 100–800, with a resolution of 120,000 at *m*/*z* 200. Tandem MS information was acquired in tMS^2^ mode with a resolution of 15,000 and higher-energy collisional dissociation collision energies (%) of 15, 30, and 45.

For **Figures 2L, M**, chromatographic separation was performed on an InertSustain C18 column (2.1 mm × 150 mm, 3 μm; GL Sciences Inc., Tokyo, Japan) with Nexera X2 UHPLC system (Shimadzu Co., Kyoto, Japan) coupled with an Exploris 120, high-performance benchtop quadrupole Orbitrap high-resolution tandem mass spectrometer (Thermo Fisher Scientific Inc., Waltham, MA) (LC/HRMS/MS). The LC separation was achieved on an InertSustain C18 column (2.1 mm × 150 mm, 3 μm; GL Sciences Inc., Tokyo, Japan) maintained at 40 °C. The mobile phases were 0.1% formic acid in water (A) and acetonitrile (B), with a flow rate of 0.30 mL/min and a gradient program as follows: 2% B (0 min); 95% B (13–20 min); and 2% B (20.1–25 min). The injection volume was 10 μL. The Full-scanning HRMS conditions were as follows: polarity, positive ionization; sheath gas, 50 arb; auxiliary gas, 10 arb; spray voltage, +3.5 kV; ion transfer temperature, 325 °C; heater temperature, 350 °C; mass resolution, 60,000; automatic gain control (AGC) target, 1 × 10^7^; maximum injection time (IT), 300 ms; and scan range, *m/z* 150–350 and 420–500. Parallel reaction monitoring (PRM) for target compounds was performed under the following conditions: mass resolution, 15,000; AGC target, 1 × 10^7^; maximum IT, 120 ms; isolation window, 0.4 Da; and normalized collision energy, 20 eV. Identification of 5-formyl THF and folic acid was performed by comparing the retention time, HRMS (mass error tolerance of 10 ppm), and HRMS/MS of the samples with those of authentic standards analyzed under identical conditions. Identification and relative quantification of folate species were performed using Cascade ver. 1.1 software (Reifycs Inc., Tokyo, Japan).

### 4.10 Measurement of tumor-killing activity *in vitro*

1×10^6^ *Traj33*^–/–^ splenocytes were stimulated in 24-well plates pre-coated with 10 μg/mL of anti-CD3ϵ (clone 2C11, Proteintech) (RRID: AB_2918368) and 2 μg/ml of anti-CD28 (clone PV1, BD Pharmingen) (RRID: AB_2795036) antibodies overnight. After stimulation, cells were retrovirally transduced with 6C2 or 12F12 TCRs following the protocol as previously described (69). 5×10^4^ *Traj33*^–/–^ splenocytes retrovirally transduced with 6C2 or 12F12 TCRs were co-cultured with 5×10^4^ CT26.mMR1 cells for 24 hours at 37 °C in a CO_2_incubator. After the co-culture, supernatants were collected for measurement of LDH released by non-viable cells using the LDH-Glo**™** Cytotoxicity Assay Kit (Promega). Following the manufacturer**’**s protocol, percent cytotoxicity was calculated using the following formula: % Cytotoxicity = (Experimental LDH release – Control group without T cells)**/**(Maximum LDH release control – Control group without T cells).

### 4.11 Statistical analyses

Statistical significance was calculated using Prism software (GraphPad, San Diego, CA) (RRID: SCR_002798). Differences with values for *p* < 0.05 were considered statistically significant.

## Data Availability

The datasets presented in this study can be found in online repositories. The names of the repository/repositories and accession number(s) can be found below: https://www.ncbi.nlm.nih.gov/geo/, GSE275014 https://www.ncbi.nlm.nih.gov/geo/, GSE249305 https://repository.massbank.jp, MPST000129 https://repository.massbank.jp, MPST000130.
