# Supplementary material for: Mucosal-associated invariant T cells recognize a tumor-derived metabolite in the DNA synthesis pathway
Source: Front Immunol. 2026 Jun 24;17:1797918. doi: 10.3389/fimmu.2026.1797918 (PMC13342176; doi:10.3389/fimmu.2026.1797918)
Supplement: Supplementary file 1 [file DataSheet1.pdf]

Supplementary Material

1 Supplementary Figures and Tables

1.1 Supplementary Figures

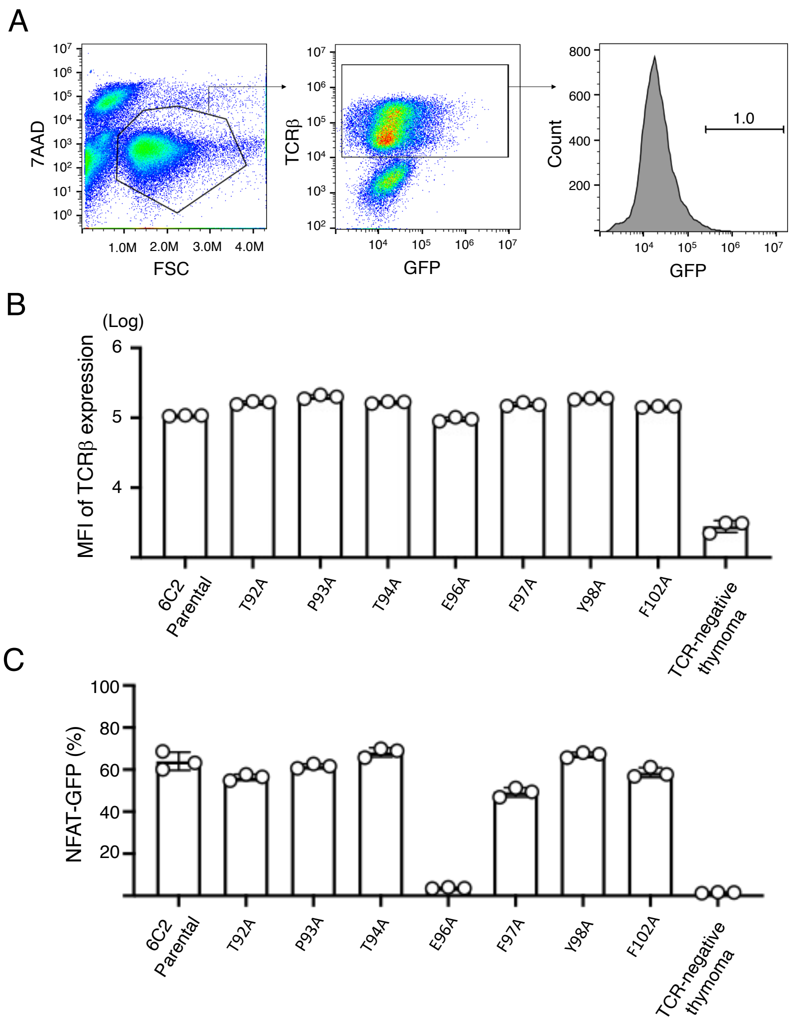

Supplementary Fig. 1. Establishment of 6C2-derived mutants.
(A) Gating strategies of flow cytometric analysis. After 6C2 was cultured with NIH3T3.mMR1 cells in the presence of anti-MR1 blocking antibody for 24 hours, cells were analyzed by flow cytometry. After excluding 7AAD+ dead cells (left), TCRb+ reporter cell lines were gated (middle) to show GFP expression as a histogram (right). **(B)** Mean fluorescence intensity (MFI) of TCRb+ expressions in 6C2 (parental cell line), its mutants and murine TCR-negative thymoma is shown. **(C)** NFAT-GFP expressions of 6C2, its mutants and murine TCR-negative thymoma were analyzed using flow cytometry after co-culture with NIH3T3.mMR1 cells. **(B, C)** Data are means ± SEM from three biological replicates per group. Data are representative of two independent experiments.

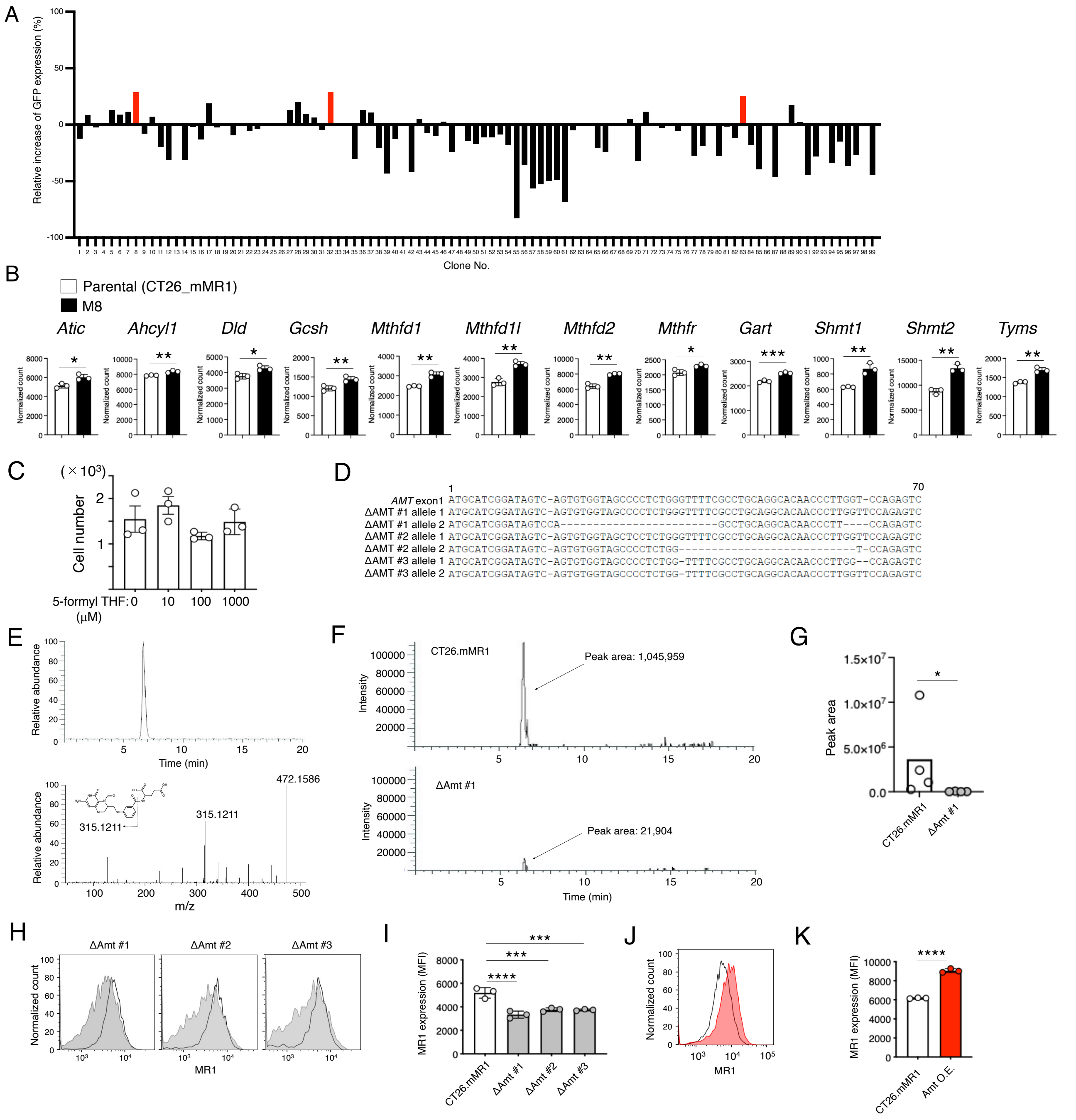

Supplementary Fig. 2. Identification of a tumor associated MAIT cell ligand.
(A)NFAT-GFP expressions in 6C2 cells were analyzed using flow cytometry after co-culture with its mutants (1–99). CT26.mMR1 cells were used as a control. The formula of relative change of NFAT-GFP expression is Relative increase of GFP expression (%) = (NFAT-GFP expression value of 6C2 with each mutant – NFAT-GFP expression value of 6C2 with CT26.mMR1 cells) / (NFAT-GFP expression value of 6C2 with CT26.mMR1 cells) × 100. **(B)** After uploading read count data, differential expression analysis was performed, employing criteria of fold change (FC) ≥ 1 and false discovery rate (FDR) < 0.1. Graphs show normalized counts of genes in parental strain CT26.mMR1 cells and M8 cells. Data are means ± SEM from three biological replicates per group. *Atic:5-*

aminoimidazole-4-carboxamide ribonucleotide formyltransferase/IMP cyclohydrolase, Ahcyl1:S-adenosylhomocysteine hydrolase-like 1, Dld:dihydrolipoamide dehydrogenase, Gcsh:glycine cleavage system protein H (aminomethyl carrier), Mthfd1: methylenetetrahydrofolate dehydrogenase (NADP+ dependent), methenyltetrahydrofolate cyclohydrolase, formyltetrahydrofolate synthase, Mthfd1l:Methylenetetrahydrofolate dehydrogenase (NADP+ dependent) 1-like, Mthfd2:Methylenetetrahydrofolate dehydrogenase (NAD+ dependent), methenyltetrahydrofolate cyclohydrolase, Mthfr: Methylenetetrahydrofolate reductase, Gart:Phosphoribosylglycinamide formyltransferase, Shmt1:Serine hydroxymethyltransferase 1 (soluble), Shmt2:Serine hydroxymethyltransferase 2 (mitochondrial), Tyms: Thymidylate synthase. **(C)** After incubation of CT26.mMR1 cells (1×10^3 cells per well) in the presence of indicated concentrations of 5-formyl THF, cell number was examined. Data are means ± SEM from three biological replicates per group. **(D)** Wild-type sequences aligned with generated mutant alleles are shown. **(E)** Chromatogram (upper) and MS/MS spectra (lower) of 5-formyl THF in the negative ion mode. **(F)** Chromatograms of metabolites from CT26.mMR1 and ΔAmt #1 cells. **(E, F)** Peaks were shown after filtration by values of MS 472.1515 and MSMS 315.1212. **(G)** Graph shows peak areas of 5-formyl THF. Data are means from four biological replicates per group. **(H-K)** Representative histograms **(H, J)** or Bar graphs **(I, K)** show surface MR1 expressions on CT26.mMR1 cells (parental), its Amt-deficient mutants (ΔAmt #1-3) **(H, I)** and Amt-overexpressing CT26.mMR1 cells (AMT O.E.) **(J, K)**. Data are means ± SEM from three biological replicates per group. **(B, G, I, K)** Statistical significance was determined by one-way ANOVA, followed by Dunnett's multiple comparison test **(I)** or unpaired two-tailed Student's t-test (B, G, K) (* $p < 0.05$, ** $p < 0.01$, *** $p < 0.001$, **** $p < 0.0001$). **(H–K)** Data are representative of two independent experiments.

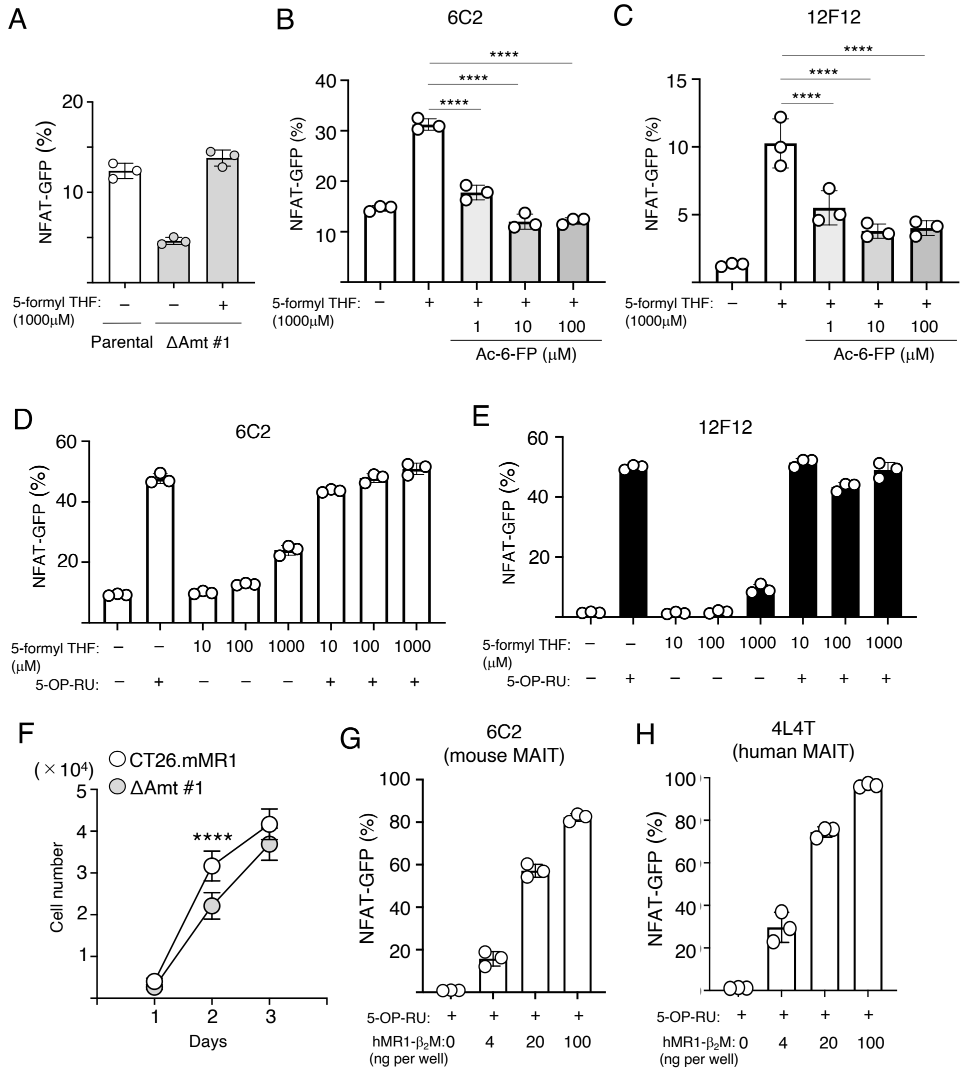

Supplementary Fig. 3. 5-formyl THF-dependent MAIT cell agonistic activity.
(A) NFAT-GFP expressions of 6C2 were analyzed after co-culture with CT26.mMR1 cells upon stimulation with or without 5-formyl THF. Data are means ± SEM from three biological replicates per group. **(B-E)** NFAT-GFP expressions of 6C2 **(B, D)** or 12F12 **(C, E)** were analyzed using flow cytometry after co-culture with CT26.mMR1 cells upon stimulation with 5-formyl THF (1000 μM) in the presence of Ac-6-FP or 5-OP-RU (10 nM). Data are means ± SEM from three biological replicates per group. **(F)** After incubation of CT26.mMR1 and ΔAmt #1 cells (1×10^3 cells per well), cell numbers were compared at each time point. Data are means ± SEM from five biological replicates at each time point. **(G, H)** NFAT-GFP expressions of 6C2 (mouse MAIT) and 4L4T (human MAIT) were analyzed after incubation with plates coated with indicated doses of human MR1-β2M complex in the presence of 5-OP-RU (10 nM). Data are means ± SEM from three biological replicates per group. **(B, C, F)** Statistical significance was determined by one-way ANOVA, followed by Dunnett's multiple comparison test **(B, C)** and two-way ANOVA followed by Bonferroni's multiple-comparison test **(F)** (*** $p < 0.001$, **** $p < 0.0001$). Data are representative of two independent experiments.

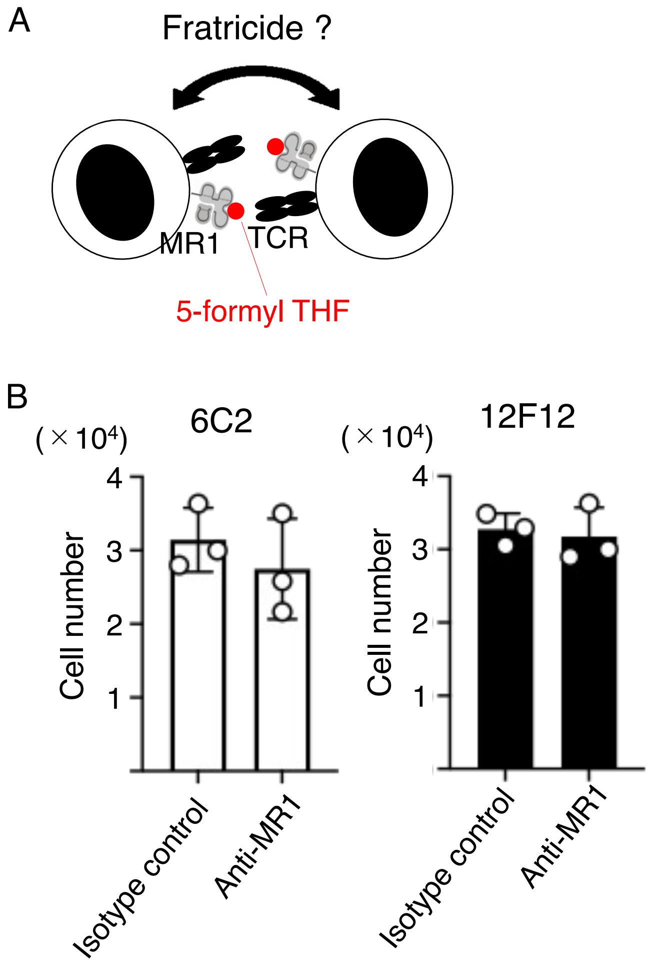

Supplementary Fig. 4. 6C2 and 12F12 mouse MAIT TCR-overexpressing T cells do not undergo fratricide upon stimulation with 5-formyl THF.
(A) Illustration of fratricide between MAIT TCR-overexpressing mouse T cells through endogenous MR1. The graphics were created with BioRender.com (https://www.biorender.com). **(B)** 6C2 and 12F12 mouse MAIT TCR-overexpressing mouse T cells from BALB/c-background *Traj33*$^{-/-}$ mice were incubated with 1000 mM of 5-formyl THF in the presence of anti-MR1 blocking antibody (25 µg/mL) or its isotype control. After incubation, 6C2 and 12F12 mouse MAIT TCR-overexpressing mouse T cells were stained with a mouse 5-OP-RU-loaded MR1 tetramer (mMR1-Tet/5-OP-RU). The mMR1-Tet/5-OP-RU^{+} cells were analyzed by flow cytometry after gating on 7AAD-negative cells. Bar graphs show viable mMR1-Tet/5-OP-RU^{+} cell numbers. Data are means ± SEM from three biological replicates per group. Data are representative of two independent experiments.

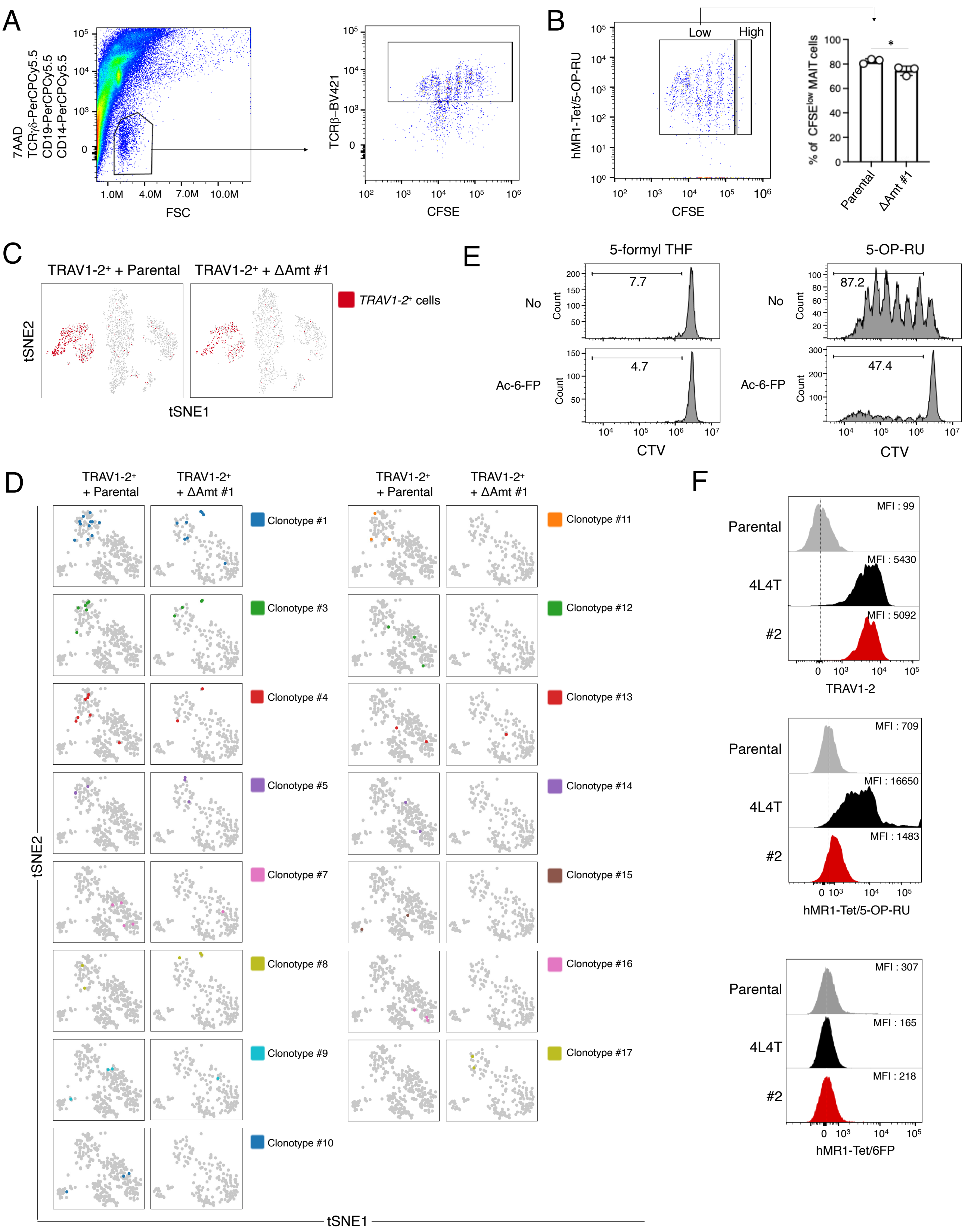

A
7AAD
TCRγδ-PerCPCy5.5
CD19-PerCPCy5.5
CD14-PerCPCy5.5
FSC
TCRβ–BV421
CFSE
B
hMR1-Tet/5-OP-RU
Low
High
CFSE
% of CFSE^low MAIT cells
Parental
ΔAmt #1
C
TRAV1-2+ + Parental
TRAV1-2+ + ΔAmt #1
TRAV1-2+ cells
tSNE2
tSNE1
E
5-formyl THF
5-OP-RU
No
Ac-6-FP
Count
7.7
4.7
87.2
47.4
CTV
D
TRAV1-2+ + Parental
TRAV1-2+ + ΔAmt #1
Clonotype #1
Clonotype #3
Clonotype #4
Clonotype #5
Clonotype #7
Clonotype #8
Clonotype #9
Clonotype #10
Clonotype #11
Clonotype #12
Clonotype #13
Clonotype #14
Clonotype #15
Clonotype #16
Clonotype #17
tSNE2
tSNE1
F
Parental
4L4T
#2
MFI : 99
MFI : 5430
MFI : 5092
TRAV1-2
MFI : 709
MFI : 16650
MFI : 1483
hMR1-Tet/5-OP-RU
MFI : 307
MFI : 165
MFI : 218
hMR1-Tet/6FP

Supplementary Fig. 5. Single-cell analysis of human MAIT cells.

(A) Gating strategy for CFSE-labeled human MAIT cells after co-culture with tumors. After excluding $7AAD^{+}$ $TCRgd^{+}$ $CD19^{+}$ $CD14^{+}$ cells, cells were plotted using CFSE fluorescence and an anti-TCRb antibody. **(B)** Bar graph shows the percentage of CFSE-low proliferating cells in hMR1-Tet/5-OP-RU^{+} cells. PBMCs from the same donor were used for all experiments. Data are means ± SEM from three biological replicates per group. Statistical significance was determined by unpaired two-tailed Student's *t*-test (* $p < 0.05$). **(C)** t-SNE plots generated by data based on single-cell transcriptome analysis. *TRAV1-2*$^{+}$ clusters were identified using Loupe Cell Browser provided by 10x Genomics (https://support.10xgenomics.com/single-cell-gene-expression/software/downloads/latest#loupetab). **(D)** t-SNE plots generated by data based on single cell transcriptome analysis. After TCR data were projected to the t-SNE plots, clonotypes were assigned. **(E)** CTV-labeled human PBMCs were stimulated with 5-formyl THF (1000mM) or 5-OP-RU (10nM) for seven days in the presence or absence of Ac-6-FP (100mM). After the culture, cells were analyzed after gating as in **(A)**. **(F)** Histograms show cells positive for TRAV1-2 (left) and, cells positive for 5-OP-RU-loaded or 6FP-loaded human MR1 tetramers. Numbers in the plots indicate mean fluorescence intensity (MFI). **(E, F)** Data are representative of two independent experiments.

1.2 Supplementary Tables

Supplementary Data file 1. Upregulated genes in M8.

Ensemble Gene ID	Gene Name	Folate synthetic pathway
ENSMUSG00000001211	1-acylglycerol-3-phosphate O-acyltransferase 3(Agpat3)	
ENSMUSG00000057134	2-aminoethanethiol dioxygenase(Ado)	
ENSMUSG00000035376	3-hydroxyacyl-CoA dehydratase 2(Hacd2)	
ENSMUSG00000022186	3-oxoacid CoA transferase 1(Oxct1)	
ENSMUSG00000053398	3-phosphoglycerate dehydrogenase(Phgdh)	
ENSMUSG00000032615	5',3'-nucleotidase, mitochondrial(Nt5m)	
ENSMUSG00000017176	5'-nucleotidase, cytosolic IIIB(Nt5c3b)	
ENSMUSG00000026192	5-aminoimidazole-4-carboxamide ribonucleotide formyltransferase/IMP cyclohydrolase(Atic)	○
ENSMUSG00000026773	6-phosphofructo-2-kinase/fructose-2,6-biphosphatase 3(Pfkfb3)	
ENSMUSG00000031807	6-phosphogluconolactonase(Pgls)	
ENSMUSG00000025428	ATP synthase F1 subunit alpha(Atp5f1a)	
ENSMUSG00000025393	ATP synthase F1 subunit beta(Atp5f1b)	
ENSMUSG00000016252	ATP synthase F1 subunit epsilon(Atp5f1e)	
ENSMUSG00000006057	ATP synthase membrane subunit c locus 1(Atp5mc1)	
ENSMUSG00000018770	ATP synthase membrane subunit c locus 3(Atp5mc3)	
ENSMUSG00000038690	ATP synthase membrane subunit f(Atp5mf)	
ENSMUSG00000038023	ATPase, H+ transporting, lysosomal V0 subunit A2(Atp6v0a2)	
ENSMUSG00000024121	ATPase, H+ transporting, lysosomal V0 subunit C(Atp6v0c)	
ENSMUSG00000013160	ATPase, H+ transporting, lysosomal V0 subunit D1(Atp6v0d1)	
ENSMUSG00000006273	ATPase, H+ transporting, lysosomal V1 subunit B2(Atp6v1b2)	
ENSMUSG00000022295	ATPase, H+ transporting, lysosomal V1 subunit C1(Atp6v1c1)	
ENSMUSG00000004285	ATPase, H+ transporting, lysosomal V1 subunit F(Atp6v1f)	
ENSMUSG00000019087	ATPase, H+ transporting, lysosomal accessory protein 1(Atp6ap1)	
ENSMUSG00000030682	CDP-diacylglycerol--inositol 3-phosphatidyltransferase(Cdipt)	
ENSMUSG00000004099	DNA methyltransferase 1(Dnmt1)	
ENSMUSG00000031168	EBP cholestenol delta-isomerase(Ebp)	
ENSMUSG00000006390	ELOVL fatty acid elongase 1(Elovl1)	
ENSMUSG00000041220	ELOVL fatty acid elongase 6(Elovl6)	
ENSMUSG00000038372	GDP-mannose 4, 6-dehydratase(Gmds)	
ENSMUSG00000037580	GTP cyclohydrolase 1(Gch1)	
ENSMUSG00000054008	N-deacetylase/N-sulfotransferase (heparan glucosaminyl) 1(Ndst1)	
ENSMUSG00000031349	NAD(P) dependent steroid dehydrogenase-like(Nsdhl)	
ENSMUSG00000025968	NADH:ubiquinone oxidoreductase core subunit S1(Ndufs1)	
ENSMUSG00000013593	NADH:ubiquinone oxidoreductase core subunit S2(Ndufs2)	
ENSMUSG00000005510	NADH:ubiquinone oxidoreductase core subunit S3(Ndufs3)	
ENSMUSG00000021606	NADH:ubiquinone oxidoreductase core subunit S6(Ndufs6)	
ENSMUSG00000059734	NADH:ubiquinone oxidoreductase core subunit S8(Ndufs8)	
ENSMUSG00000016427	NADH:ubiquinone oxidoreductase subunit A1(Ndufa1)	
ENSMUSG00000026260	NADH:ubiquinone oxidoreductase subunit A10(Ndufa10)	
ENSMUSG00000036199	NADH:ubiquinone oxidoreductase subunit A13(Ndufa13)	
ENSMUSG00000014294	NADH:ubiquinone oxidoreductase subunit A2(Ndufa2)	
ENSMUSG00000030869	NADH:ubiquinone oxidoreductase subunit AB1(Ndufab1)	
ENSMUSG00000037601	NME/NM23 nucleoside diphosphate kinase 1(Nme1)	
ENSMUSG00000032478	NME/NM23 nucleoside diphosphate kinase 6(Nme6)	
ENSMUSG00000029632	Ndufa4, mitochondrial complex associated(Ndufa4)	
ENSMUSG00000027893	S-adenosylhomocysteine hydrolase-like 1(Ahcyl1)	○
ENSMUSG00000026603	SET and MYND domain containing 2(Smyd2)	
ENSMUSG00000001750	T cell, immune regulator 1, ATPase, H+ transporting, lysosomal V0 protein A3(Tcirg1)	
ENSMUSG00000050796	UDP-Gal:betaGal beta 1,3-galactosyltransferase, polypeptide 6(B3galt6)	
ENSMUSG00000026956	UDP-N-acteylglucosamine pyrophosphorylase 1-like 1(Uap1l1)	
ENSMUSG00000029201	UDP-glucose dehydrogenase(Ugdh)	
ENSMUSG00000007036	abhydrolase domain containing 16A(Abhd16a)	
ENSMUSG00000029482	acetoacetyl-CoA synthetase(Aacs)	
ENSMUSG00000028405	aconitase 1(Aco1)	
ENSMUSG00000022477	aconitase 2, mitochondrial(Aco2)	
ENSMUSG00000024981	acyl-CoA synthetase long-chain family member 5(Acsl5)	
ENSMUSG00000021226	acyl-CoA thioesterase 2(Acot2)	
ENSMUSG00000028937	acyl-CoA thioesterase 7(Acot7)	
ENSMUSG00000006589	adenine phosphoribosyl transferase(Aprt)	
ENSMUSG00000027889	adenosine monophosphate deaminase 2(Ampd2)	
ENSMUSG00000028792	adenylate kinase 2(Ak2)	
ENSMUSG00000039058	adenylate kinase 5(Ak5)	
ENSMUSG00000078941	adenylate kinase 6(Ak6)	
ENSMUSG00000022407	adenylosuccinate lyase(Adsl)	
ENSMUSG00000074207	alcohol dehydrogenase 1 (class I)(Adh1)	
ENSMUSG00000055301	alcohol dehydrogenase 7 (class IV), mu or sigma polypeptide(Adh7)	
ENSMUSG00000025007	aldehyde dehydrogenase 18 family, member A1(Aldh18a1)	
ENSMUSG00000053279	aldehyde dehydrogenase family 1, subfamily A1(Aldh1a1)	
ENSMUSG00000001642	aldo-keto reductase family 1 member B(Akr1b1)	
ENSMUSG00000036820	amidohydrolase domain containing 2(Amdhd2)	
ENSMUSG00000042082	arylsulfatase B(Arsb)	
ENSMUSG00000029752	asparagine synthetase(Asns)	
ENSMUSG00000030268	branched chain aminotransferase 1, cytosolic(Bcat1)	
ENSMUSG00000013629	carbamoyl-phosphate synthetase 2, aspartate transcarbamylase, and dihydroorotase(Cad)	
ENSMUSG00000027555	carbonic anhydrase 13(Car13)	
ENSMUSG00000027357	cardiolipin synthase 1(Crls1)	
ENSMUSG00000000326	catechol-O-methyltransferase(Comt)	
ENSMUSG00000027035	ceramide synthase 6(Cers6)	
ENSMUSG00000024843	choline kinase alpha(Chka)	
ENSMUSG00000034807	collagen beta(1-O)galactosyltransferase 1(Colgalt1)	
ENSMUSG00000022742	coproporphyrinogen oxidase(Cpox)	
ENSMUSG00000021947	crystallin, lambda 1(Cryl1)	
ENSMUSG00000023044	cysteine sulfinic acid decarboxylase(Csad)	
ENSMUSG00000028633	cytidine 5'-triphosphate synthase 1(Ctps1)	
ENSMUSG00000020638	cytidine/uridine monophosphate kinase 2(Cmpk2)	
ENSMUSG00000031818	cytochrome c oxidase subunit 4I1(Cox4i1)	
ENSMUSG00000000088	cytochrome c oxidase subunit 5A(Cox5a)	
ENSMUSG00000061518	cytochrome c oxidase subunit 5B(Cox5b)	
ENSMUSG00000041697	cytochrome c oxidase subunit 6A1(Cox6a1)	
ENSMUSG00000014313	cytochrome c oxidase subunit 6C(Cox6c)	
ENSMUSG00000031231	cytochrome c oxidase subunit 7B(Cox7b)	
ENSMUSG00000035885	cytochrome c oxidase subunit 8A(Cox8a)	
ENSMUSG00000022551	cytochrome c-1(Cyc1)	
ENSMUSG00000031562	dCMP deaminase(Dctd)	
ENSMUSG00000022210	dehydrogenase/reductase 4(Dhrs4)	
ENSMUSG00000027068	dehydrogenase/reductase 9(Dhrs9)	
ENSMUSG00000038633	delta 4-desaturase, sphingolipid 1(Degs1)	
ENSMUSG00000026281	deoxythymidylate kinase(Dtymk)	
ENSMUSG00000039497	dermatan sulfate epimerase(Dse)	
ENSMUSG00000040479	diacylglycerol kinase zeta(Dgkz)	
ENSMUSG00000004789	dihydrolipoamide S-succinyltransferase(Dlst)	
ENSMUSG00000020664	dihydrolipoamide dehydrogenase(Dld)	○
ENSMUSG00000026810	dolichyl-phosphate mannosyltransferase subunit 2, regulatory(Dpm2)	
ENSMUSG00000029687	enhancer of zeste 2 polycomb repressive complex 2 subunit(Ezh2)	
ENSMUSG00000063524	enolase 1, alpha non-neuron(Eno1)	
ENSMUSG00000059040	enolase 1B, retrotransposed(Eno1b)	
ENSMUSG00000029326	enolase-phosphatase 1(Enoph1)	
ENSMUSG00000025465	enoyl Coenzyme A hydratase, short chain, 1, mitochondrial(Echs1)	
ENSMUSG00000022040	epoxide hydrolase 2, cytoplasmic(Ephx2)	
ENSMUSG00000010663	fatty acid desaturase 1(Fads1)	
ENSMUSG00000024665	fatty acid desaturase 2(Fads2)	
ENSMUSG00000040170	flavin containing monooxygenase 2(Fmo2)	
ENSMUSG00000009566	folylpolyglutamyl synthetase(Fpgs)	
ENSMUSG00000031266	galactosidase, alpha(Gla)	
ENSMUSG00000021003	galactosylceramidase(Galc)	
ENSMUSG00000034707	glucosamine (N-acetyl)-6-sulfatase(Gns)	
ENSMUSG00000052102	glucosamine-6-phosphate deaminase 1(Gnpda1)	
ENSMUSG00000091387	glucosaminyl (N-acetyl) transferase 4, core 2 (beta-1,6-N-acetylglucosaminyltransferase)(Gcnt4)	
ENSMUSG00000025534	glucuronidase, beta(Gusb)	
ENSMUSG00000028124	glutamate-cysteine ligase, modifier subunit(Gclm)	
ENSMUSG00000031700	glutamic pyruvate transaminase (alanine aminotransferase) 2(Gpt2)	
ENSMUSG00000026615	glutamyl-prolyl-tRNA synthetase 1(Eprs1)	
ENSMUSG00000025068	glutathione S-transferase omega 1(Gsto1)	
ENSMUSG00000063856	glutathione peroxidase 1(Gpx1)	
ENSMUSG00000075706	glutathione peroxidase 4(Gpx4)	
ENSMUSG00000031584	glutathione reductase(Gsr)	
ENSMUSG00000006378	glycine C-acetyltransferase (2-amino-3-ketobutyrate-coenzyme A ligase)(Gcat)	
ENSMUSG00000034424	glycine cleavage system protein H (aminomethyl carrier)(Gcsh)	○
ENSMUSG00000003865	glycogen synthase 1, muscle(Gys1)	
ENSMUSG00000042148	heme A:farnesyltransferase cytochrome c oxidase assembly factor 10(Cox10)	
ENSMUSG00000025745	hydroxyacyl-CoA dehydrogenase trifunctional multienzyme complex subunit alpha(Hadha)	
ENSMUSG00000027195	hydroxysteroid (17-beta) dehydrogenase 12(Hsd17b12)	
ENSMUSG00000074797	inosine triphosphatase (nucleoside triphosphate pyrophosphatase)(Itpa)	
ENSMUSG00000021385	inositol 1,3,4,5,6-pentakisphosphate 2-kinase(Ippk)	
ENSMUSG00000060733	inositol polyphosphate multikinase(Ipmk)	
ENSMUSG00000039682	leucine aminopeptidase 3(Lap3)	
ENSMUSG00000015889	leukotriene A4 hydrolase(Lta4h)	
ENSMUSG00000030725	lipoyl(octanoyl) transferase 2 (putative)(Lipt2)	
ENSMUSG00000019179	malate dehydrogenase 2, NAD (mitochondrial)(Mdh2)	
ENSMUSG00000048755	malonyl CoA:ACP acyltransferase (mitochondrial)(Mcat)	
ENSMUSG00000008763	mannosidase, alpha, class 1A, member 2(Man1a2)	
ENSMUSG00000036620	mannoside acetylglucosaminyltransferase 4, isoenzyme B(Mgat4b)	
ENSMUSG00000005667	methylenetetrahydrofolate dehydrogenase (NAD+ dependent), methenyltetrahydrofolate cyclohydrolase(Mthfd2)	○
ENSMUSG00000040675	methylenetetrahydrofolate dehydrogenase (NADP+ dependent) 1-like(Mthfd1l)	○
ENSMUSG00000021048	methylenetetrahydrofolate dehydrogenase (NADP+ dependent), methenyltetrahydrofolate cyclohydrolase, formyltetrahydrofolate synthase(Mthfd1)	○
ENSMUSG00000029009	methylenetetrahydrofolate reductase(Mthfr)	○
ENSMUSG00000031604	methylsterol monoxygenase 1(Msmo1)	
ENSMUSG00000041939	mevalonate kinase(Mvk)	
ENSMUSG00000025037	monoamine oxidase A(Maoa)	
ENSMUSG00000030269	myotubularin related protein 14(Mtmr14)	
ENSMUSG00000031918	myotubularin related protein 2(Mtmr2)	
ENSMUSG00000032271	nicotinamide N-methyltransferase(Nnmt)	
ENSMUSG00000029310	nudix hydrolase 9(Nudt9)	
ENSMUSG00000011179	ornithine decarboxylase, structural 1(Odc1)	
ENSMUSG00000005615	phosphate cytidylyltransferase 1, choline, alpha isoform(Pcyt1a)	
ENSMUSG00000000301	phosphatidylethanolamine N-methyltransferase(Pemt)	
ENSMUSG00000017721	phosphatidylinositol glycan anchor biosynthesis, class T(Pigt)	
ENSMUSG00000021518	phosphatidylserine synthase 1(Ptdss1)	
ENSMUSG00000059173	phosphodiesterase 1A, calmodulin-dependent(Pde1a)	
ENSMUSG00000027513	phosphoenolpyruvate carboxykinase 1, cytosolic(Pck1)	
ENSMUSG00000040618	phosphoenolpyruvate carboxykinase 2 (mitochondrial)(Pck2)	
ENSMUSG00000043445	phosphoglycolate phosphatase(Pgp)	
ENSMUSG00000022711	phosphomannomutase 2(Pmm2)	
ENSMUSG00000029246	phosphoribosyl pyrophosphate amidotransferase(Ppat)	
ENSMUSG00000020899	phosphoribosylformylglycinamidine synthase (FGAR amidotransferase)(Pfas)	
ENSMUSG00000022962	phosphoribosylglycinamide formyltransferase(Gart)	○
ENSMUSG00000024640	phosphoserine aminotransferase 1(Psat1)	
ENSMUSG00000029446	phosphoserine phosphatase(Psph)	
ENSMUSG00000003131	platelet-activating factor acetylhydrolase, isoform 1b, subunit 2(Pafah1b2)	
ENSMUSG00000005447	platelet-activating factor acetylhydrolase, isoform 1b, subunit 3(Pafah1b3)	
ENSMUSG00000020520	polypeptide N-acetylgalactosaminyltransferase 10(Galnt10)	
ENSMUSG00000032527	propionyl Coenzyme A carboxylase, beta polypeptide(Pccb)	
ENSMUSG00000026820	prostaglandin E synthase 2(Ptges2)	
ENSMUSG00000068417	purine-nucleoside phosphorylase 2(Pnp2)	
ENSMUSG00000115338	purine-nucleoside phosphorylase(Pnp)	
ENSMUSG00000024892	pyruvate carboxylase(Pcx)	
ENSMUSG00000021748	pyruvate dehydrogenase (lipoamide) beta(Pdhb)	
ENSMUSG00000010914	pyruvate dehydrogenase complex, component X(Pdhx)	
ENSMUSG00000030978	ribonucleotide reductase M1(Rrm1)	
ENSMUSG00000040364	secretory blood group 1(Sec1)	
ENSMUSG00000026662	selenophosphate synthetase 1(Sephs1)	
ENSMUSG00000049091	selenophosphate synthetase 2(Sephs2)	
ENSMUSG00000033735	sepiapterin reductase(Spr)	
ENSMUSG00000020534	serine hydroxymethyltransferase 1 (soluble)(Shmt1)	○
ENSMUSG00000025403	serine hydroxymethyltransferase 2 (mitochondrial)(Shmt2)	○
ENSMUSG00000006442	spermidine synthase(Srm)	
ENSMUSG00000022351	squalene epoxidase(Sqle)	
ENSMUSG00000009863	succinate dehydrogenase complex, subunit B, iron sulfur (Ip)(Sdhb)	
ENSMUSG00000039231	suppressor of variegation 3-9 1(Suv39h1)	
ENSMUSG00000026646	suppressor of variegation 3-9 2(Suv39h2)	
ENSMUSG00000025574	thymidine kinase 1(Tk1)	
ENSMUSG00000022615	thymidine phosphorylase(Tymp)	
ENSMUSG00000025747	thymidylate synthase(Tyms)	○
ENSMUSG00000030884	ubiquinol cytochrome c reductase core protein 2(Uqcrc2)	
ENSMUSG00000038462	ubiquinol-cytochrome c reductase, Rieske iron-sulfur polypeptide 1(Uqcrfs1)	
ENSMUSG00000020163	ubiquinol-cytochrome c reductase, complex III subunit XI(Uqcr11)	
ENSMUSG00000022814	uridine monophosphate synthetase(Umps)	
ENSMUSG00000026558	uridine-cytidine kinase 2(Uck2)	
ENSMUSG00000024066	xanthine dehydrogenase(Xdh)	

Supplementary Data file 2. Human TCR clonotypes identified by single cell analysis.

Clonotype #	TCRα	CDR3α	TCRβ	CDR3β	# of clonotypes with parental tumor cell	# of clonotypes with ΔAmt #1
1	*TRAV1-2/TRAJ33*	CAVTDSNYQLIW	*TRBV20-1/TRBJ2-1/TRBC2, TRBV20-1/TRBJ2-1/TRBC2*	CSARDREFSYNEQFF, CSASVGEGASYNEQF	11	8
2	*TRAV1-2/TRAJ33*	CAATDSNYQLIW	*TRBV6-1/TRBJ2-1/TRBC2*	CASSPRTEAYNEQFF	11	0
3	*TRAV1-2/TRAJ33*	CAVKDSNYQLIW	*TRBV6-1/TRBJ2-1/TRBC2*	CASSEEEGTYNEQFF	6	4
4	*TRAV1-2/TRAJ33*	CAVRDSNYQLIW	*TRBV6-1/TRBD1/TRBJ1-1/TRBC1*	CASSEDESGAQEAFF	7	2
5	*TRAV1-2/TRAJ33*	CAVRDSNYQLIW	*TRBV4-3/TRBJ2-3/TRBC2*	CASSQELAGGPDTQYF	3	3
6	*TRAV1-2/TRAJ12*	CAVMDSSYKLIF	*TRBV20-1/TRBD2/TRBJ2-3/TRBC2*	CSARKLAGGDSDTQYF	6	0
7	*TRAV1-2/TRAJ33*	CAVRDSNYQLIW	*TRBV15/TRBJ2-1/TRBC2*	CATSSDAGGGYDEQFF	4	1
8	*TRAV1-2/TRAJ33*	CAVVDSNYQLIW	*TRBV4-3/TRBD2/TRBJ2-1/TRBC2*	CASSQELAGGYNEQFF	2	3
9	*TRAV1-2/TRAJ33*	CAALDSNYQLIW	*TRBV20-1/TRBJ1-6/TRBC1, TRBV20-1/TRBJ2-1/TRBC2*	CSAALVPGTDNSPLHF, CSASEGDSYNEQFF	4	1
10	*TRAV1-2/TRAJ33*	CAVLDSNYQLIW	*TRBV15/TRBJ2-5/TRBC2*	CATSGGGEQETQYF	3	0
11	*TRAV1-2/TRAJ33*	CAVRDGNYQLIW	*TRBV6-5/TRBJ2-7/TRBC2*	CASSYGVAGSYEQYF	3	0
12	*TRAV1-2/TRAJ33*	CAFMDSNYQLIW	*TRBV6-1/TRBJ2-7/TRBC2*	CASSESAGTSYEQYF	3	0
13	*TRAV1-2/TRAJ33*	CAAMDSNYQLIW	*TRBV20-1/TRBJ1-6/TRBC1*	CSAALVPGTDNSPLHF	2	1
14	*TRAV1-2/TRAJ33*	CAGMDSNYQLIW	*TRBV4-2/TRBJ2-3/TRBC2*	CASSQKLAGGPDTQYF	2	0
15	*TRAV1-2/TRAJ33*	CAAMDSNYQLIW	*TRBV6-1/TRBJ2-7/TRBC2*	CASSYGARGEQYF	2	0
16	*TRAV1-2/TRAJ33*	CAASDSNYQLIW	*TRBV20-1/TRBJ2-4/TRBC2*	CSATDRDPNIQYF	3	0
17	*TRAV1-2/TRAJ33*	CAVRDSNYQLIW	*TRBV4-2/TRBJ1-3/TRBC1, TRBV6-2/TRBJ2-7/TRBC2*	CASSQDREGVSGNTIYF, CASSYGDTSSYEQ	0	2

Supplementary Data file 3. Antibodies used in this study.

Antigen name	Reactivity	Fluorescence	Manufacturer	Clone name
CD3ε	human	FITC	Bioegend	UCHT1
CD161	human	FITC	Biolgend	HP-3G10
TRAV1-2	human	PE	Bioegend	3C10
CD137	human	PE	Bioegend	4B4-1
CD19	human	PerCP–Cy5.5	Bioegend	HIB19
TCRγδ	human	PerCP–Cy5.5	Bioegend	B1
CD14	human	PerCP–Cy5.5	Bioegend	63D3
CD69	human	BV421	Bioegend	FN50
TCRβ	mouse	BV421	Bioegend	H57-597
MR1	human/mouse	PE	Bioegend	26.5

Supplementary Data file 4. Healthy donors enrolled in this study.

Case #	Age	Gender	Ethnicity
1	32	female	East Asian ethnic group
2	29	female	East Asian ethnic group
3	25	female	East Asian ethnic group
4	30	male	East Asian ethnic group
